# Entropy optimization and response surface methodology of blood hybrid nanofluid flow through composite stenosis artery with magnetized nanoparticles (Au-Ta) for drug delivery application

**DOI:** 10.1038/s41598-023-36931-6

**Published:** 2023-06-17

**Authors:** Ebrahem A. Algehyne, N. Ameer Ahammad, Mohamed E. Elnair, Mohamed Zidan, Yasir Y. Alhusayni, B. O. El-Bashir, Anwar Saeed, Ali Saleh Alshomrani, Faris Alzahrani

**Affiliations:** 1 Department of Mathematics, Faculty of Science, University of Tabuk, P.O.Box.741, Tabuk, 71491 Saudi Arabia; 2 Department of Physics, Faculty of Science, University of Tabuk, P.O.Box.741, Tabuk, 71491 Saudi Arabia; 3 Centre of Excellence in Theoretical and Computational Science (TaCS-CoE), Faculty of Science, King Mongkut’s University of Technology, Thonburi (KMUTT), Bangkok, Thailand; 4 Mathematical Modelling and Applied Computation Research Group (MMAC), Department of Mathematics, King Abdul Aziz University, Jeddah, Saudi Arabia

**Keywords:** Fluid dynamics, Applied mathematics

## Abstract

Entropy creation by a blood-hybrid nanofluid flow with gold-tantalum nanoparticles in a tilted cylindrical artery with composite stenosis under the influence of Joule heating, body acceleration, and thermal radiation is the focus of this research. Using the Sisko fluid model, the non-Newtonian behaviour of blood is investigated. The finite difference (FD) approach is used to solve the equations of motion and entropy for a system subject to certain constraints. The optimal heat transfer rate with respect to radiation, Hartmann number, and nanoparticle volume fraction is calculated using a response surface technique and sensitivity analysis. The impacts of significant parameters such as Hartmann number, angle parameter, nanoparticle volume fraction, body acceleration amplitude, radiation, and Reynolds number on the velocity, temperature, entropy generation, flow rate, shear stress of wall, and heat transfer rate are exhibited via the graphs and tables. Present results disclose that the flow rate profile increase by improving the Womersley number and the opposite nature is noticed in nanoparticle volume fraction. The total entropy generation reduces by improving radiation. The Hartmann number expose a positive sensitivity for all level of nanoparticle volume fraction. The sensitivity analysis revealed that the radiation and nanoparticle volume fraction showed a negative sensitivity for all magnetic field levels. It is seen that the presence of hybrid nanoparticles in the bloodstream leads to a more substantial reduction in the axial velocity of blood compared to Sisko blood. An increase in the volume fraction results in a noticeable decrease in the volumetric flow rate in the axial direction, while higher values of infinite shear rate viscosity lead to a significant reduction in the magnitude of the blood flow pattern. The blood temperature exhibits a linear increase with respect to the volume fraction of hybrid nanoparticles. Specifically, utilizing a hybrid nanofluid with a volume fraction of 3% leads to a 2.01316% higher temperature compared to the base fluid (blood). Similarly, a 5% volume fraction corresponds to a temperature increase of 3.45093%.

## Introduction

The first law of thermodynamics is concerned exclusively with the quantity of energy and its transformation across forms; it makes no distinction between these two aspects. When designing a process for usage in the real world, engineers are primarily concerned with maintaining the quality and rate of energy deterioration. The development of entropy reduces the quality of energy, as stated by the second law of thermodynamics, which stipulates that the amount of energy is maintained throughout a real system but the quality of energy must decline and is measured by entropy. Examining the distribution of entropy creation within the flow field is crucial for mitigating this loss in energy quality (the exergy) in a fluid flow problem. Minimizing entropy production during fluid flow with heat transfer is a well-researched problem. The amount of energy that cannot be converted into work is expressed in thermodynamics as entropy. Two examples of practical entropy generation are heat exchanger pumps and electronic cooling systems. The development of methods to stop the loss of priceless energy is of great interest to scientists and engineers, especially in thermodynamical systems where it may result in severe disturbance. Heat is transported by radiation, conduction, convection, and evaporation in the human body^1–4^. Furthermore, energy is transported through the circulatory system, where pulmonary blood flow loses heat to surrounding tissues^5–7^. When temperatures drop below 20 °C, the human body loses heat through conduction and radiation. In order to regulate this scenario, entropy production is crucial in limiting the wastage of valuable energy. Bejan^8^ demonstrated the features of thermal conductivity in fluids through entropy optimization. Zaman et al.^9^ studied the effect magnetohydrodynamics and entropy production have on pulsatile blood flow in a curved stenosed w-shaped channel. The results demonstrate that the value of the Bejan number is significantly affected by increasing the curvature parameter. Entropy production in blood hydromagnetic flow via a curved permeable artery with stenosis was studied in depth by Kumawat et al.^10^, taking into account the effects of a heat source, changing viscosity, and chemical reaction.

Computer simulations and physical–mathematical models of non-Newtonian fluids have received a lot of interest among researchers. Non-Newtonian fluid classifications contain grease, medications, industrial lubricants, gels, chemicals (polymers, paints, and plastics), foodstuffs (honey, yogurt, and ketchup), and ecological systems such as highly concentrated sediments, oil spills, mudflows, and pollutant discharge^11^. Non-Newtonian liquids are employed in wide range of industrial processes, including the drawing of plastic films, petroleum purification, food technology, chemical materials, coating and dielectric material manufacture, aerospace, metal spinning activities, and paper production. The standard Navier–Stokes equations for the viscous model are insufficient for non-Newtonian fluids because of their basic properties. The researchers proposed various nonlinear mathematical models, such as the Williamson model, Carreau-Yasuda, Casson model, Tangent hyperbolic^12^, Bingham plastic, Maxwell model, Oldroyd-B, Eyring-Powell^13^, Brinkman type and Jeffrey fluid to describe the precise nature of non-Newtonian fluids. Medium to high shear rates are well modelled by the Sisko fluid model. By selecting various material parameters, it may show numerous common traits of Newtonian or non-Newtonian fluids. Eid et al.^14^ analysed the nonlinear thermal radiation-assisted boundary-layer flow and heat transfer of a Sisko blood-based bio-fluid towards a nonlinearly stretched surface. Xingting et al.^15^ studied the finite volume approach, which was used to predict the thermal behaviour of blood flow in arteries with different radii and stenosis angles using the non-Newtonian Sisko model. Bhatti et al.^16^ examined the endoscopic study on peristaltic blood flow of Sisko fluid containing titanium magnetic nanoparticles through a uniform tube. The outcomes revealed that the pressure gradient increases for higher values of the Hartmann number and Sisko fluid parameter. Ali et al.^17^ explored the pulsating blood flow in a narrowing stenotic artery using the Sisko fluid model. Zaman et al.^18^ examined the two-phase, pulsatile, non-steady hemodynamic blood flow through an artery while the body was under acceleration using the Sisko model. Apparently, the material parameter of the Sisko model has a greater effect on coronary artery blood flow than it does on femoral artery blood flow.

Nanotechnology has drawn a lot of interest from scientists and engineers since it is employed in a variety of real-world applications, such as the biological, heat transfer^19–24^ and medical sectors. Ferrite, silver, gold and copper nanoparticles are among the many nanoparticles that are used in proteins, nucleic acids, medication delivery, genes and vaccines. As a result of their high levels of biocompatibility, magnetic, chemical, special mechanical and thermal efficiency^25–31^. With superior quenching efficiencies, imaging, targeting ligands, probes, and considerable surface modifiability, gold nanoparticles (atomic number = 79) are widely used in medical applications for Ribonucleic acid (RNA) measurement (through optical biosensors) and treatment of malignant tumours. Furthermore, gold nanoparticles have no harmful effects in living organisms. Gold nanoparticles' unique optical property increases their utility in a wide range of therapeutic (including radiation for destroying cancer cells and photothermal) and diagnostic (including optical imaging, cell imaging, and computed tomography) settings. Gold nanoparticles may be functionalized in amazing ways using DNA, proteins, antibodies, polyelectrolytes, and ligands^32^. They are also used for medication shipping. In this study, blood containing gold-tantalum nanoparticles is examined via the lens of arterial stenosis. It is rather typical to have atherosclerotic plaques or arterial stenosis, which is a constriction of the human artery system. The usual blood flow pattern of the artery is disturbed by stenosis. A certain amount of blood flow is necessary for our kidneys to remove waste. Several injuries and an increase in blood pressure occur as a result of artery constriction, which prevents the normal amount of rich oxygenated blood from reaching our kidneys^33^. Abdelsalam et al.^34^ analysed the hemodynamic of blood flowing through arteries with aneurysms and stenosis while immersed in a fractional second-grade Au-Al2O3 hybrid nanofluid subjected to electroosmotic impact. Dolui et al.^35^ examined the dynamics of three types of nanofluids flowing through arteries with composite stenosis: mono(gold), a hybrid (copper–gold) and a tri-hybrid (copper–gold-silver). On the other hand, Tantalum (Ta) is an excellent choice when there is a need for high corrosion resistance. While it may not be among the most superior metals, it exhibits favourable chemical resistance properties. Additionally, its body-centred cubic crystal structure enables easy operation at temperatures below ambient conditions. However, it is important to note that due to its high reactivity, tantalum nanoparticles (Ta-NPs) are susceptible to oxidation in the presence of air. The use of nanoscale tantalum has found diverse applications, including enhancing capacitor efficiency and reducing circuit size, thereby enabling the creation of smaller circuits and compact packaging. In the field of medicine, planar tantalum is well-known for its remarkable biocompatibility, comparable to other therapeutic metallic materials, making it a popular choice in clinical settings. Efforts are being made to expand the biomedical applications of tantalum, particularly in utilizing tantalum NPs as metallic photoacoustic agents for tumour scanning^36^ and as contrast agents in multi-energy imaging applications^37^ and X-ray absolute scattering tests^38^.

The practical importance of nano-drug delivery methods prompted the present investigation. Managing the treatment choice process for arterial disease is also a key focus of this numerical simulation. Numerical blood flow simulation holds significant promise in supporting decision-making processes for the treatment of cardiovascular diseases. While the conventional approach to treat stenosis involves the use of stents or catheters placed inside the affected artery, there is a growing trend towards targeted delivery of nano-drugs to specific locations. This approach also initiates the formation of blood clots at the narrowed portion of the artery, and computational simulations can be employed to predict the impact of these post-treatment processes. Several studies have looked at blood flow in stenosis arteries with different shapes, sizes, and other characteristics, as shown by the aforementioned research. Nonetheless, there has been no research on the sensitivity analysis and entropy generation of Sisko blood hybrid nanofluid flow with gold-tantalum nanoparticles in a stenotic artery. Additional applications for gold-tantalum nanoparticles include drug delivery, diagnosis of cancer, treatment of cardiovascular disease, and chemotherapy. This article aims to delve further into the transport of non-Newtonian pharmacodynamics in realistic physiological geometries. With a focus on unsteady Sisko blood flow, the study investigates the properties of hybrid nanoparticles (gold and tantalum) as they traverse composite symmetric stenosis within an affected artery. Owing to these drivers, the present framework is centred on the flow of a Sisko hybrid nanofluid made of bio-magnetic gold and tantalum blood through a composite stenosis artery, complete with time-dependent sensitivity analysis and the generation of entropy with joule heating, viscous dissipation, and the Lorentz force. By replacing identical variables with non-similar ones, the dimensional flow equations become dimensionless. In order to solve the simplified flow equations, FDM is employed. Graphs depict the physical parameters impacts that are derived from the regime equations.

## Mathematical formulation

**Figure 1:**
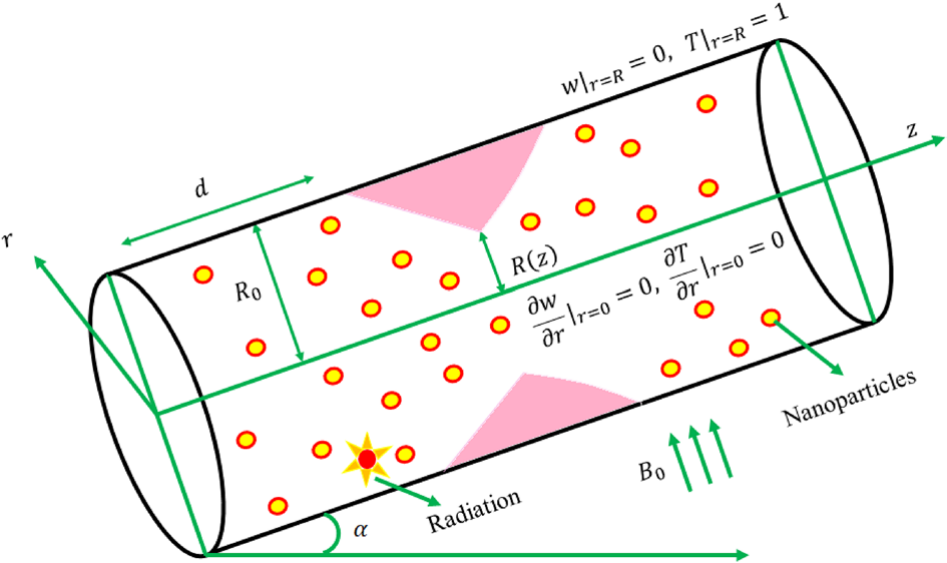
Schematic view of the present model.

This exploration considers the flow of blood via a cylindrical artery with composite stenosis under the conditions of pulsatile, incompressible, time-dependent, and laminar flow. Besides, blood flow in the human coronary artery is modelled using a bidirectional rheological blood flow model, and blood is doped with nanoparticles. In Fig. 1, the physical arrangement is shown in cylindrical coordinates $\left(r,\theta ,z\right)$^36,37^, where the radial coordinate $\left(r\right)$, the circumferential (azimuthal) coordinate $\left( \theta \right)$ and the axial (longitudinal) coordinate $\left(z\right)$ represent the corresponding axes The biomagnetic blood flow is examined using the nanoparticle volume fraction model. It should be emphasized that the biomagnetic blood fluid contains gold and tantalum nanoparticles, and both the basic fluid (blood) and the nanomaterials are listed in Table 1 with their respective thermophysical properties (gold and tantalum). Flow of blood goes against the direction of greatest Lorentz force.

**Table 1:** Thermo-physical properties of *Au-Ta/Blood*^25–27^.

Physical properties	Blood	Au	Ta
$\rho \; \left(\frac{\mathrm{kg}}{{\mathrm{m}}^{3}}\right)$	1050	10,500	16,650
${C}_{p} \; \left(\frac{\mathrm{J}}{\mathrm{kg} \; \mathrm{K}}\right)$	3617	235	686.2
$k \; \left(\frac{\mathrm{W}}{\mathrm{m} \,\mathrm{K}}\right)$	0.52	429	0.52
$\gamma \, \left(\frac{1}{\mathrm{K}}\right)$	0.18	0.0000142	0.0000063
$\sigma \left(\mathrm{S}/\mathrm{m}\right)$	1.33	4.5 $\times {10}^{7}$	7.7 $\times {10}^{6}$

Dimensional form of stenosis with composite^38^

1 $$\left.\begin{array}{ll}{R}_{0}-2\delta \left(\overline{z }-\overline{d }\right)\left(\frac{1}{{l}_{0}}\right), & \quad \overline{d }\le \overline{z }\le \overline{d }+0.5{l}_{0}\\ {R}_{0}-0.5\delta \left(1+\mathrm{cos}\left(2\pi \frac{1}{{l}_{0}}\right)\left(\overline{z }-\overline{d }-0.5{l}_{0}\right)\right), & \quad \overline{d }+0.5{l}_{0}<\overline{z }<\overline{d }+{l}_{0}\\ {R}_{0}, & \quad \text{otherwise}\end{array}\right\}=R\left(z\right)$$

where, $\delta$, $\overline{z }$, $l_{0}$, ${R}_{0}$ and $\overline{d }$ are height of the stenosis, axial coordinate, stenosis total length, healthy artery radius and distance from the stenosis origin, respectively.

This model assumes that the time-dependent, bidirectional blood flows in the artery; hence, the flow field become

2 $$T=T\left(r,z,t\right), V=\left[\overline{u }\left(r,z,t\right),0,\overline{w }\left(r,z,t\right)\right].$$

Using the above-described flow considerations, the blood transport equations are expressed in cylindrical coordinates as follows^39–42^

3 $$\frac{\partial \overline{u} }{\partial \overline{r} }+\frac{\overline{u} }{\overline{r} }+\frac{\partial \overline{w} }{\partial \overline{z} }=0,$$

4 $$\begin{aligned}{\rho }_{hnf}\left(\frac{\partial \overline{u} }{\partial \overline{t} }+\overline{u }\frac{\partial \overline{u} }{\partial \overline{r} }+\overline{w }\frac{\partial \overline{u} }{\partial \overline{z} }\right) & =-\frac{\partial P}{\partial \overline{r} }+{\mu }_{hnf}\left(\frac{1}{\overline{r}}\frac{\partial }{\partial \overline{r} }\left(\overline{r}{S }_{\overline{r }\overline{r} }\right)+\frac{\partial }{\partial \overline{z} }\left({S}_{\overline{r }\overline{z} }\right)\right)\\ & \quad +g{\left(\gamma \rho \right)}_{hnf}\left(T-{T}_{1}\right)cos\alpha \end{aligned}$$

5 $$\begin{aligned} {\rho }_{hnf}\left(\frac{\partial \overline{w} }{\partial \overline{t} }+\overline{u }\frac{\partial \overline{w} }{\partial \overline{r} }+\overline{w }\frac{\partial \overline{w} }{\partial \overline{z} }\right) & =-\frac{\partial P}{\partial \overline{z} }+{\mu }_{hnf}\left(\frac{1}{\overline{r}}\frac{\partial }{\partial \overline{r} }\left(\overline{r}{S }_{\overline{r }\overline{z} }\right)+\frac{\partial }{\partial \overline{z} }\left({S}_{\overline{z }\overline{z} }\right)\right)\\ \\ & \quad +g{\left(\gamma \rho \right)}_{hnf}\left(T-{T}_{1}\right)sin\alpha +{\rho }_{hnf}G\left(t\right)-{\sigma }_{hnf}{B}_{0}^{2}\overline{w} \end{aligned}$$

6 $$\begin{aligned}{\left(\rho {C}_{p}\right)}_{hnf}\left(\frac{\partial T}{\partial \overline{t} }+\overline{u}\frac{\partial T }{\partial \overline{r} }+\overline{w}\frac{\partial T }{\partial \overline{z} }\right) & ={k}_{hnf}\left(\frac{{\partial }^{2}T}{\partial {\overline{r} }^{2}}+\frac{1}{\overline{r}}\frac{\partial T }{\partial \overline{r} }+\frac{{\partial }^{2}T}{\partial {\overline{z} }^{2}}\right)-\frac{\partial {q}_{r}}{\partial \overline{r} }\\ & \quad +{\mu }_{hnf}\left[{S}_{\overline{r }\overline{r} }\frac{\partial \overline{u} }{\partial \overline{r} }+{S}_{\overline{r }\overline{z} }\frac{\partial \overline{w} }{\partial \overline{r} }+{S}_{\overline{z }\overline{r} }\frac{\partial \overline{w} }{\partial \overline{z} }+{S}_{\overline{z }\overline{z} }\frac{\partial \overline{w} }{\partial \overline{z} }\right]+{\sigma }_{hnf}{B}_{0}^{2}{\overline{w} }^{2}\end{aligned}$$

The following boundary and beginning conditions apply to the study of geometry^37^.

7 $$\left.\begin{array}{c}\overline{w }\left(\overline{r },0\right)=0,\frac{\partial \overline{w }\left(0,t\right)}{\partial \overline{r} }=0,\overline{w }\left(R,t\right)=0,\\ T\left(\overline{r },0\right)=0,\frac{\partial T\left(0,t\right)}{\partial \overline{r} }=0,T\left(R,t\right)={T}_{w}.\end{array}\right\}$$

where $T, { \beta }_{nf}, {\sigma }_{nf}, g, t, {B}_{0}, {\left({C}_{p}\right)}_{nf}, {\rho }_{nf}, P, {k}_{nf}, \alpha$ and ${\mu }_{nf}$ are the temperature, thermal expansion, electrical conductivity, time, gravitational acceleration, uniform magnetic field, specific heat capacity, density, pressure, thermal conductivity, angle parameter and dynamic viscosity, respectively. The Cauchy stress tensor T for Sisko fluid can be defined as^43^ ${\rm T}=S-pI$, where $S$ and $pI$ are the deviatoric and spherical parts of ${\rm T}$ respectively. Extra stress tensor for the Sisko fluid is $S={A}_{1}\left[\mu {\left({\Pi }^{2}\right)}^{\frac{n-1}{2}}+{\mu }_{\infty }\right]$. $\Pi =\sqrt{\left(\frac{1}{2}\right)trac{\left({A}_{1}\right)}^{2}}$ is the rate of strain $B$ is the material constant, ${\mu }_{\infty }$ is the infinite-shear-rate viscosity and $\mu$ dynamic viscosity of blood, where ${A}_{1}={\left(\nabla V\right)}^{T}+\left(\nabla V\right)$ is the rate of deformation tensor, and the constitutive relation of the Sisko model in yields is as follows^15^

8 $$\left.\begin{array}{c}{S}_{\overline{r }\overline{r} }=2\left(\frac{\partial \overline{u} }{\partial \overline{r} }\right)\left\{{\mu }_{\infty }+\mu {\left|{\left(\frac{\partial \overline{u} }{\partial \overline{z} }+\frac{\partial \overline{w} }{\partial \overline{r} }\right)}^{2}+2\left({\left(\frac{\partial \overline{u} }{\partial \overline{r} }\right)}^{2}+{\left(\frac{\overline{u} }{\overline{r} }\right)}^{2}+{\left(\frac{\partial \overline{w} }{\partial \overline{z} }\right)}^{2}\right)\right|}^{\frac{n-1}{2}}\right\},\\ {S}_{\overline{z }\overline{z} }=2\frac{\partial \overline{w} }{\partial \overline{z}}\left\{{\mu }_{\infty }+\mu {\left|{\left(\frac{\partial \overline{u} }{\partial \overline{z} }+\frac{\partial \overline{w} }{\partial \overline{r} }\right)}^{2}+2\left({\left(\frac{\partial \overline{u} }{\partial \overline{r} }\right)}^{2}+{\left(\frac{\overline{u} }{\overline{r} }\right)}^{2}+{\left(\frac{\partial \overline{w} }{\partial \overline{z} }\right)}^{2}\right)\right|}^{\frac{n-1}{2}}\right\},\\ {S}_{\overline{r }\overline{z} }=\left(\frac{\partial \overline{u} }{\partial \overline{z} }+\frac{\partial \overline{w} }{\partial \overline{r} }\right)\left\{{\mu }_{\infty }+\mu {\left|{\left(\frac{\partial \overline{u} }{\partial \overline{z} }+\frac{\partial \overline{w} }{\partial \overline{r} }\right)}^{2}+2\left({\left(\frac{\partial \overline{u} }{\partial \overline{r} }\right)}^{2}+{\left(\frac{\overline{u} }{\overline{r} }\right)}^{2}+{\left(\frac{\partial \overline{w} }{\partial \overline{z} }\right)}^{2}\right)\right|}^{\frac{n-1}{2}}\right\}.\end{array}\right\}$$

Radiative heat flow $\left({q}_{r}\right)$ is computed using the Rosseland diffusion approximation^40^ to simulate thermal radiation ${q}_{r}=-\frac{4}{3}\frac{{\sigma }^{*}}{{k}_{e}^{*}}\left(\frac{\partial {T}^{4}}{\partial r}\right)$, where ${k}_{e}^{*}$ and ${\sigma }^{*}$ denote the mean absorption coefficient and the Stefan-Boltzmann constant, respectively.

The non-dimensional quantities are given as^39–43^:

9 $$r=\frac{\overline{r}}{{R }_{0}},z=\frac{\overline{z}}{{l }_{0}},p=\frac{{R}_{0}^{2}P}{{U}_{0}{l}_{0}{\mu }_{f}},u=\frac{{l}_{0}\overline{u}}{{\delta }^{*}{U}_{0}},\theta =\frac{T-{T}_{1}}{{T}_{w}-{T}_{1}},{S}_{rr}=\frac{{l}_{0}}{{U}_{0}{\mu }_{f}}{S}_{\overline{r }\overline{r}} {S }_{zz}=\frac{{l}_{0}}{{U}_{0}{\mu }_{f}}{S}_{\overline{z }\overline{z} },{S}_{rz}=\frac{{R}_{0}}{{U}_{0}{\mu }_{f}}{S}_{\overline{r }\overline{z} },w=\frac{\overline{w}}{{U }_{0}},R=\frac{\overline{R}}{{R }_{0}},t=\frac{{U}_{0}\overline{t}}{{R }_{0}},d=\frac{\overline{d}}{{l }_{0}}.$$

As a result of merging the above-defined variables and disregarding the bars, the governing equations are stated in a non-dimensionalized form, as shown below

10 $$\delta \left(\frac{\partial u}{\partial r}+\frac{u}{r}+\frac{1}{\delta }\frac{\partial w}{\partial z}\right)=0,$$

11 $$\begin{aligned}{\text{R}}_{\text{e}}\left(\frac{{\rho }_{hnf}}{{\rho }_{f}}\right)\delta {\varepsilon }^{2}\left(\Lambda \frac{\partial u}{\partial t}+\left(\delta \varepsilon \right)u\frac{\partial u}{\partial r}+\varepsilon w\frac{\partial u}{\partial z}\right) & =-\frac{\partial p}{\partial r}+\left(\frac{{\mu }_{hnf}}{{\mu }_{f}}\right){\left(\frac{{R}_{0}}{{l}_{0}}\right)}^{2}\left(\frac{1}{r}\frac{\partial }{\partial r}\left(r{S}_{rr}\right)+\frac{\partial }{\partial z}\left({S}_{rz}\right)\right)\\ & \quad +\left(\frac{{\left(\rho \gamma \right)}_{hnf}}{{\left(\rho \gamma \right)}_{f}}\right)\varepsilon cos\left(\alpha \right){G}_{r}\theta ,\end{aligned}$$

12 $$\begin{aligned}{\text{R}}_{\text{e}}\left(\frac{{\rho }_{hnf}}{{\rho }_{f}}\right)\left(\Lambda \frac{\partial w}{\partial t}+\left(\delta \varepsilon \right)u\frac{\partial w}{\partial r}+\varepsilon w\frac{\partial w}{\partial z}\right) & =-\frac{\partial p}{\partial z}+\left(\frac{{\mu }_{hnf}}{{\mu }_{f}}\right)\left(\frac{1}{r}\frac{\partial }{\partial r}\left(r{S}_{rz}\right)+{\left(\frac{{R}_{0}}{{l}_{0}}\right)}^{2}\frac{\partial }{\partial z}\left({S}_{zz}\right)\right)\\ & \quad +\left(\frac{{\rho }_{hnf}}{{\rho }_{f}}\right)G\left(t\right)+\left(\frac{\left(\rho \gamma \right)_{hnf}}{{\left(\rho \gamma \right)}_{f}}\right){G}_{r}\theta sin\left(\alpha \right)-\frac{{\sigma }_{hnf}}{{\sigma }_{f}}{M}_{a}^{2}w,\end{aligned}$$

13 $$\begin{array}{c}\left(\frac{{\left(\rho {C}_{p}\right)}_{hnf}}{{\left(\rho {C}_{p}\right)}_{f}}\right)\left(\frac{\partial \theta }{\partial t}+\left(\delta \varepsilon \right)u\frac{\partial \theta }{\partial r}+\varepsilon w\frac{\partial \theta }{\partial z}\right)=\frac{1}{\mathrm{Pr}{R}_{e}}\left(\frac{{k}_{hnf}}{{k}_{f}}\right)\left(\frac{{\partial }^{2}\theta }{\partial {r}^{2}}+\frac{1}{r}\frac{\partial \theta }{\partial r}+{\varepsilon }^{2}\frac{{\partial }^{2}\theta }{\partial {z}^{2}}\right)+\frac{{N}_{R}}{\mathrm{Pr}{R}_{e}}\frac{{\partial }^{2}\theta }{\partial {r}^{2}}\\ +\left(\frac{{\sigma }_{hnf}}{{\sigma }_{f}}\right)\frac{{E}_{C}{M}_{a}^{2}}{{\mathrm{R}}_{e}}{w}^{2}+\left(\frac{{\mu }_{hnf}}{{\mu }_{f}}\right)\frac{{E}_{C}}{{R}_{e}}\left(\delta {\left(\frac{{R}_{0}}{{l}_{0}}\right)}^{2}{S}_{rr}\frac{\partial u}{\partial r}+{S}_{rz}\frac{\partial w}{\partial r}+{\left(\frac{{R}_{0}}{{l}_{0}}\right)}^{2}\left(\delta {S}_{zr}\frac{\partial u}{\partial z}+{S}_{zz}\frac{\partial w}{\partial z}\right)\right).\end{array}$$

$$\left.\begin{array}{c}{S}_{rr}=\left(\varepsilon \delta \frac{\partial u}{\partial r}\right)\left\{m+{\left|{\left(\delta \frac{\partial u}{\partial z}+\frac{\partial w}{\partial r}\right)}^{2}+2\left(\varepsilon \delta \left({\left(\frac{\partial u}{\partial r}\right)}^{2}+{\left(\frac{u}{r}\right)}^{2}\right)+\varepsilon {\left(\frac{\partial w}{\partial z}\right)}^{2}\right)\right|}^{\frac{n-1}{2}}\right\},\\ {S}_{zz}=\varepsilon \frac{\partial w}{\partial z}\left\{m+{\left|{\left(\delta \frac{\partial u}{\partial z}+\frac{\partial w}{\partial r}\right)}^{2}+2\left(\varepsilon \delta {\left({\left(\frac{\partial u}{\partial r}\right)}^{2}+{\left(\frac{u}{r}\right)}^{2}\right)}^{2}+\varepsilon {\left(\frac{\partial w}{\partial z}\right)}^{2}\right)\right|}^{\frac{n-1}{2}}\right\},\\ {S}_{rz}=\left(\delta \frac{\partial u}{\partial z}+\frac{\partial w}{\partial r}\right)\left\{m+{\left|{\left(\delta \frac{\partial u}{\partial z}+\frac{\partial w}{\partial r}\right)}^{2}+2\left(\varepsilon \delta \left({\left(\frac{\partial u}{\partial r}\right)}^{2}+{\left(\frac{u}{r}\right)}^{2}\right)+\varepsilon {\left(\frac{\partial w}{\partial z}\right)}^{2}\right)\right|}^{\frac{n-1}{2}}\right\}.\end{array}\right\}$$

Here, $\Lambda =\frac{{\rho }_{f}\omega {R}_{0}^{2}}{2\pi {\mu }_{f}}$ is Womersley number, ${U}_{0}$ is the reference velocity, ${\mathrm{M}}_{a}^{2}=\frac{{\sigma }_{f}{\mathrm{B}}_{0}^{2}{\mathrm{R}}_{0}^{2}}{{\mu }_{f}}$ is Hartmann number, ${G}_{r}=\frac{g{\rho }_{f}{R}_{0}^{2}{\gamma }_{f}\left({T}_{w}-{T}_{1}\right)}{{U}_{0}{\mu }_{f}}$ is Grashof number, ${\text{R}}_{\text{e}}=\frac{{U}_{0}{\rho }_{f}{R}_{0}}{{\mu }_{f}}$ is Reynolds number, ${E}_{C}=\frac{{U}_{0}^{2}}{{\left({C}_{p}\right)}_{f}\left({T}_{w}-{T}_{1}\right)}$ is Eckert number, ${T}_{w}$ is the wall temperature, ${S}_{rr}$, ${S}_{zz}$ and ${S}_{rz}$ are dimensionless extra stress tensor components, $\varepsilon =\frac{{R}_{0}}{{l}_{0}}$ is the vessel aspect ratio, $m=\frac{{\mu }_{\infty }}{\mu }$ is dimensionless infinite shear rate viscosity, ${N}_{R}=\frac{16{\sigma }_{e}{T}_{1}^{3}}{3{k}_{f}{k}_{e}}$ thermal radiation, $Pr=\frac{{\left({C}_{p}\right)}_{f}{\mu }_{f}}{{k}_{f}}$ is Prandtl number, and $\delta =\frac{{\delta }^{*}}{{R}_{0}}$ is the stenosis height parameter.

The thermos-physical nature of hybrid nanofluids is given by^44^

14 $$\left.\begin{aligned}\frac{{\mu }_{hnf}}{{\mu }_{f}}=\frac{1}{{\left(1-{\phi }_{Au}\right)}^{2.5}{\left(1-{\phi }_{Ta}\right)}^{2.5}},{\alpha }_{hnf}=\frac{{k}_{hnf}}{{\left(\rho {C}_{p}\right)}_{hnf}},\\ \frac{{\rho }_{hnf}}{{\rho }_{f}}=\left(1-{\phi }_{Ta}\right)\left(\left(1-{\phi }_{Au}\right)+{\phi }_{Au}\frac{{\rho }_{Au}}{{\rho }_{f}}\right)+{\phi }_{Ta}\frac{{\rho }_{Ta}}{{\rho }_{f}},\\ \frac{{\left(\rho {C}_{p}\right)}_{hnf}}{{\left(\rho {C}_{p}\right)}_{f}}=\left(1-{\phi }_{Ta}\right)\left(\left(1-{\phi }_{Au}\right)+{\phi }_{Au}\frac{{\left(\rho {C}_{p}\right)}_{Au}}{{\left(\rho {C}_{p}\right)}_{f}}\right)+{\phi }_{Ta}\frac{{\left(\rho {C}_{p}\right)}_{Ta}}{{\left(\rho {C}_{p}\right)}_{f}},\\ \frac{{\left(\rho \gamma \right)}_{hnf}}{{\left(\rho \gamma \right)}_{f}}=\left(1-{\phi }_{Ta}\right)\left(\left(1-{\phi }_{Au}\right)+{\phi }_{Au}\frac{{\left(\rho \gamma \right)}_{Au}}{{\left(\rho \gamma \right)}_{f}}\right)+{\phi }_{Ta}\frac{{\left(\rho \gamma \right)}_{Ta}}{{\left(\rho \gamma \right)}_{f}},\\ \frac{{k}_{hnf}}{{k}_{bf}}=\frac{\left(1+2{\phi }_{Ta}\right){k}_{Ta}+2\left(1-{\phi }_{Ta}\right){k}_{bf}}{\left(1-{\phi }_{Ta}\right){k}_{Ta}+\left(2+{\phi }_{Ta}\right){k}_{bf}},where\frac{{k}_{bf}}{{k}_{f}}=\frac{\left(1+2{\phi }_{Au}\right){k}_{Au}+2\left(1-{\phi }_{Au}\right){k}_{f}}{\left(1-{\phi }_{Au}\right){k}_{Au}+\left(2+{\phi }_{Au}\right){k}_{f}}\\ \frac{{\sigma }_{hnf}}{{\sigma }_{bf}}=\frac{{\sigma }_{Ta}+2{\sigma }_{bf}-2{\phi }_{Ta}\left({\sigma }_{bf}-{\sigma }_{Ta}\right)}{{\sigma }_{Ta}+2{\sigma }_{bf}+{\phi }_{Ta}\left({\sigma }_{bf}-{\sigma }_{Ta}\right)},{\text{w}}{\text{h}}{\text{e}}{\text{r}}{\text{e}} \, \frac{{\sigma }_{bf}}{{\sigma }_{f}}=\frac{{\sigma }_{Au}+2{\sigma }_{f}-2{\phi }_{Au}\left({\sigma }_{f}-{\sigma }_{Au}\right)}{{\sigma }_{Au}+2{\sigma }_{f}+{\phi }_{Au}\left({\sigma }_{f}-{\sigma }_{Au}\right)}.\end{aligned}\right\}$$

The axial pressure gradient that appears in Eq. (12) can be expressed as follows

15 $$-\frac{\partial p}{\partial z}={A}_{0}+{A}_{1}t\mathrm{cos}\left(2\pi {w}_{p}\right),t>0,$$

where the mean pressure gradient is represented by ${A}_{0}$ and the mean pulsatile amplitude is represented by ${A}_{1}$. The reduced form of pressure gradient by applying non similar variables is

16 $$-\frac{\partial p}{\partial z}=\left(e\mathrm{cos}\left({c}_{1}t\right)+1\right){B}_{1},$$

where, $${B}_{1}=\frac{{A}_{0}{a}^{2}}{{\mu }_{0}{U}_{0}}$$, $$e=\frac{{A}_{1}}{{A}_{0}},$$ and $${c}_{1}=\frac{2\pi a{w}_{p}}{{U}_{0}}$$.

The human body acceleration of the current model is written as

17 $$G\left(t\right)={a}_{g}\left(\mathrm{cos}\left({\omega }_{b}t+\kappa \right)\right)$$

By employing Eq. (9), the dimensionless form of Eq. (17) is becomes as follows

18 $$G\left(t\right)={B}_{2}\left(\mathrm{cos}\left({c}_{2}t+\kappa \right)\right)$$

In the above equation, $\kappa$ is the lead angle to the heart activity, ${c}_{2}=\frac{{\omega }_{b}}{\omega }$ is the body force, and ${B}_{2}={\rho }_{f}{a}_{g}\frac{{R}_{0}^{2}}{{\mu }_{f}{U}_{0}}$ is the amplitude of body acceleration.

This study is based on two assumptions, $\delta \ll 1$ and $\varepsilon = \text{O} \left(1\right)$: (i) the parameter of stenosis height is less than one, and (ii) the aspect ratio of vessel is less than one. By considering these hypotheses, the reduced form of equations is manifested as follows:

19 $$\frac{\partial w}{\partial z}=0,$$

20 $$\frac{\partial p}{\partial r}=0,$$

21 $$\begin{aligned}{\text{R}}_{\text{e}}\left(\frac{{\rho }_{hnf}}{{\rho }_{f}}\right)\left(\Lambda \frac{\partial w}{\partial t}\right) & ={B}_{1}\left(1+e\mathrm{cos}\left({c}_{1}t\right)\right)+\frac{{\mu }_{hnf}}{{\mu }_{f}}\frac{1}{r}\frac{\partial }{\partial r}\left(r\left(m+{\left(\frac{\partial w}{\partial r}\right)}^{n-1}\right)\frac{\partial w}{\partial r}\right)\\ & \quad +\left(\frac{{\rho }_{hnf}}{{\rho }_{f}}\right){B}_{2}\left(\mathrm{cos}\left({c}_{2}t+\kappa \right)\right)+\left(\frac{{\left(\rho \gamma \right)}_{hnf}}{{\left(\rho \gamma \right)}_{f}}\right){G}_{r}\theta sin\left(\alpha \right)-\frac{{\sigma }_{hnf}}{{\sigma }_{f}}{M}_{a}^{2}w,\end{aligned}$$

22 $$\begin{aligned}\left(\frac{{\left(\rho {C}_{p}\right)}_{hnf}}{{\left(\rho {C}_{p}\right)}_{f}}\right)\left(\frac{\partial \theta }{\partial t}\right)& =\frac{1}{\mathrm{Pr}{R}_{e}}\left(\frac{{k}_{hnf}}{{k}_{f}}\right)\left(\frac{{\partial }^{2}\theta }{\partial {r}^{2}}+\frac{1}{r}\frac{\partial \theta }{\partial r}\right)+\frac{{N}_{R}}{\mathrm{Pr}{R}_{e}}\frac{{\partial }^{2}\theta }{\partial {r}^{2}}\\ & \quad +\left(\frac{{\sigma }_{hnf}}{{\sigma }_{f}}\right)\frac{{E}_{C}{M}_{a}^{2}}{{\mathrm{R}}_{e}}{w}^{2}+\left(\frac{{\mu }_{hnf}}{{\mu }_{f}}\right)\frac{{E}_{C}}{{\mathrm{R}}_{e}}\left(m+{\left(\frac{\partial w}{\partial r}\right)}^{n-1}\right){\left(\frac{\partial w}{\partial r}\right)}^{2}\end{aligned}$$

The reduced form of Composite stenosis is

23 $$\left.\begin{array}{ll}1-\left(z-d\right)2{\delta }^{*}, & \quad d\le z\le d+0.5\\ 1-\left(1+\mathrm{cos}2\pi \left(z-d-0.5\right)\right)0.5{\delta }^{*}, & \quad d+0.5<z<d+1\\ 1, & \quad {\text{otherwise}}\end{array}\right\}=R\left(z\right)$$

Applying the radial coordinate form $\left(x=\frac{r}{R\left(z\right)}\right)$ to the reduced equations yields a rectangular computing domain. In view of this transformation, the final form of equations are

24 $$\begin{array}{c}{\text{R}}_{\text{e}}\left(\frac{{\rho }_{hnf}}{{\rho }_{f}}\right)\left(\Lambda \frac{\partial w}{\partial t}\right)={B}_{1}\left(1+e\mathrm{cos}\left({c}_{1}t\right)\right)+\frac{{\mu }_{hnf}}{{\mu }_{f}}\frac{1}{x{R}^{2}}\frac{\partial }{\partial x}\left(x\left(m+{\left(\frac{1}{R}\frac{\partial w}{\partial x}\right)}^{n-1}\right)\frac{\partial w}{\partial x}\right)\\ +\left(\frac{{\rho }_{hnf}}{{\rho }_{f}}\right){B}_{2}\left(\mathrm{cos}\left({c}_{2}t+\kappa \right)\right)+\left(\frac{{\left(\rho \gamma \right)}_{hnf}}{{\left(\rho \gamma \right)}_{f}}\right){G}_{r}\theta sin\left(\alpha \right)-\frac{{\sigma }_{hnf}}{{\sigma }_{f}}{M}_{a}^{2}w,\end{array}$$

25 $$\begin{array}{c}\left(\frac{{\left(\rho {C}_{p}\right)}_{hnf}}{{\left(\rho {C}_{p}\right)}_{f}}\right)\left(\frac{\partial \theta }{\partial t}\right)=\frac{1}{\mathrm{Pr}{R}_{e}}\left(\frac{{k}_{hnf}}{{k}_{f}}\right)\left(\frac{1}{{R}^{2}}\right)\left(\frac{{\partial }^{2}\theta }{\partial {x}^{2}}+\frac{1}{x}\frac{\partial \theta }{\partial x}\right)+\left(\frac{1}{{R}^{2}}\right)\frac{{N}_{R}}{\mathrm{Pr}{R}_{e}}\frac{{\partial }^{2}\theta }{\partial {x}^{2}}\\ +\left(\frac{{\sigma }_{hnf}}{{\sigma }_{f}}\right)\frac{{E}_{C}{M}_{a}^{2}}{{\mathrm{R}}_{e}}{w}^{2}+\left(\frac{{\mu }_{hnf}}{{\mu }_{f}}\right)\frac{{E}_{C}}{{\mathrm{R}}_{e}}\left(\frac{1}{{R}^{2}}\right)\left(m+{\left(\frac{1}{R}\frac{\partial w}{\partial x}\right)}^{n-1}\right){\left(\frac{\partial w}{\partial x}\right)}^{2}\end{array}$$

Subject to the limiting conditions

26 $$\left.\begin{array}{c}w\left(x,t=0\right)=0,\theta \left(x,t=0\right)=0,\\ \frac{\partial w\left(x=0,t\right)}{\partial x}=0,\frac{\partial \theta \left(x=0,t\right)}{\partial x}=0,\\ w\left(x=1,t\right)=0,\theta \left(x=1,t\right)=1.\end{array}\right\}$$

Parameters of hemodynamic, such as wall shear stress $\left({\tau }_{s}\right)$, resistance impedance $\left(\lambda \right)$, Nusselt number $\left(Nu\right)$ and volumetric flow rate $\left({Q}_{F}\right)$, are formulated mathematically as

27 $$\left.\begin{array}{c}{\tau }_{s}=\frac{1}{R\left(z\right)}{\left(\left(m+\frac{1}{{\left(R\left(z\right)\right)}^{n-1}}{\left|\frac{\partial w}{\partial x}\right|}^{n-1}\right)\frac{\partial w}{\partial x}\right)}_{x=1},\\ \lambda =L\left[\frac{\left(\frac{\partial p}{\partial z}\right)}{{Q}_{F}}\right]=L\left[\frac{{B}_{1}\left(1+e\mathrm{cos}\left(2\pi t\right)\right)}{{\left(R\left(z\right)\right)}^{2}2\pi \left(\underset{0}{\overset{1}{\int }}wxdx\right)}\right],\\ Nu=\frac{1}{R\left(z\right)}{\left(\frac{\partial \theta }{\partial x}\right)}_{x=1}\\ {Q}_{F}={\left(R\left(z\right)\right)}^{2}2\pi \left(\underset{0}{\overset{1}{\int }}wxdx\right).\end{array}\right\}$$

## Entropy generation

Entropy generation serves as a measure for quantifying the rate of irreversibility’s within a given process. When confronted with constraints in the heat transport system, the second law of thermodynamics assumes a more prominent role compared to the first law. The dimensional representation for volumetric entropy generation can be expressed as follows^7,10^:

28 $${E}_{g}=\frac{{k}_{f}}{{T}_{1}^{2}}\left[\frac{{k}_{hnf}}{{k}_{f}}+\frac{16{\sigma }_{e}{T}_{1}^{3}}{3{k}_{f}{k}_{e}}\right]\left({\left(\frac{\partial T}{\partial \overline{r} }\right)}^{2}+{\left(\frac{\partial T}{\partial \overline{z} }\right)}^{2}\right) +\frac{{\mu }_{hnf}}{{T}_{1}}\left[{S}_{\overline{r }\overline{r} }\frac{\partial \overline{u} }{\partial \overline{r} }+{S}_{\overline{r }\overline{z} }\frac{\partial \overline{w} }{\partial \overline{r} }+{S}_{\overline{z }\overline{r} }\frac{\partial \overline{w} }{\partial \overline{z} }+{S}_{\overline{z }\overline{z} }\frac{\partial \overline{w} }{\partial \overline{z} }\right] +\frac{{\sigma }_{hnf}{B}_{0}^{2}{\overline{w} }^{2}}{{T}_{1}}$$

The utilization of dimensionless variables in Eq. (9) and assumptions $(\delta \ll 1$ and $\varepsilon = \text{O} \left(1\right))$: leads us to derive the following expression for the entropy equation.

29 $${E}_{g}=\frac{{k}_{f}{\left({T}_{w}-{T}_{1}\right)}^{2}}{{T}_{1}^{2}{R}_{0}^{2}}\left[\frac{{k}_{hnf}}{{k}_{f}}+\frac{16{\sigma }_{e}{T}_{1}^{3}}{3{k}_{f}{k}_{e}}\right]\left({\left(\frac{\partial \theta }{\partial r}\right)}^{2}\right)+\frac{{\mu }_{hnf}{U}_{0}^{2}}{{T}_{1}{R}_{0}^{2}}\left[{S}_{rz}\frac{\partial w}{\partial r}\right]+\frac{{\sigma }_{hnf}{B}_{0}^{2}{U}_{0}^{2}{R}_{0}^{2}{w}^{2}}{{T}_{1}}$$

The dimensionless entropy generation number, denoted as ${N}_{g}=\frac{{T}_{1}^{2}{R}_{0}^{2}}{{k}_{f}{\left({T}_{w}-{T}_{1}\right)}^{2}}{E}_{g}$, represents the ratio between the change in the rate of generated entropy and the characteristic entropy transfer. Equation (29) can be employed to express this relationship with radial transformation

30 $$\begin{aligned} {N}_{g} & =\left(\frac{{k}_{hnf}}{{k}_{f}}+{N}_{R}\right)\left(\frac{1}{R}\right)\left(\frac{\partial \theta }{\partial x}\right)^2+\frac{{\mu }_{hnf}}{{\mu }_{f}}\left(\frac{Br}{\eta }\right)\left(\frac{1}{R}\right)\left(m+{\left(\left(\frac{1}{R}\right)\frac{\partial w}{\partial x}\right)}^{n-1}\right){\left(\frac{\partial w}{\partial x}\right)}^{2}\\ & \quad +\left(\frac{{\sigma }_{hnf}}{{\sigma }_{f}}\right)\frac{Br{M}_{a}^{2}}{\eta }{w}^{2}\end{aligned}$$

where, $\eta =\frac{{T}_{w}-{T}_{1}}{{T}_{1}}$, temperature difference, ${N}_{g}=\frac{{T}_{1}^{2}{R}_{0}^{2}}{{k}_{f}{\left({T}_{w}-{T}_{1}\right)}^{2}}{E}_{g}$ dimensionless entropy generation and $Br=\frac{{U}_{0}^{2} {\mu }_{f}}{{k}_{f}\left({T}_{w}-{T}_{1}\right)}$ Brickman number.

## Finite difference simulation

A variety of numerical methods for solving partial differential equations are explored in Hoffmann's book^45^. Additionally, the book demonstrates the differentiation between parabolic, elliptic, and hyperbolic forms of partial differential equations. This book recommends using the explicit finite difference method when dealing with time-dependent issues. As a result, current time-dependent reduced Eqs. (18) and (23) are computed by employing the explicit finite difference method. This simulation adheres to the stability and convergence requirements by setting the smaller time and spatial variable. Following this notation are the steps necessary to derive the finite difference formulation of several partial derivatives:

$$\frac{\partial w}{\partial x}={w}_{x}\cong \frac{{w}_{i+1}^{k}-{w}_{i-1}^{k}}{2\Delta x},$$ $$\frac{{\partial }^{2}w}{\partial {x}^{2}}={w}_{xx}\cong \frac{{w}_{i+1}^{k}-2{w}_{i}^{k}+{w}_{i-1}^{k}}{{\left(\Delta x\right)}^{2}},$$ and $$\frac{\partial w}{\partial t}\cong \frac{{w}_{i}^{k+1}-2{w}_{i}^{k}-{w}_{i}^{k}}{\Delta t}.$$

Similarly, $$\frac{\partial \theta }{\partial x}={\theta }_{x}\cong \frac{{\theta }_{i+1}^{k}-{\theta }_{i-1}^{k}}{2\Delta x},$$ $$\frac{{\partial }^{2}\theta }{\partial {x}^{2}}={\theta }_{xx}\cong \frac{{\theta }_{i+1}^{k}-2{\theta }_{i}^{k}+{\theta }_{i-1}^{k}}{{\left(\Delta x\right)}^{2}},$$ and $$\frac{\partial \theta }{\partial t}\cong \frac{{\theta }_{i}^{k+1}-2{\theta }_{i}^{k}-{\theta }_{i}^{k}}{\Delta t}.$$

Using the aforementioned derivative equations, (24) & (25) are easily simplified to the following form:

31 $${w}_{j}^{i+1}={w}_{j}^{i}+\frac{\Delta t}{\mathrm{Re}\Lambda \left(\frac{{\rho }_{hnf}}{{\rho }_{f}}\right)}\left[\begin{array}{c}{B}_{1}\left(1+e\mathrm{cos}\left({c}_{1}{t}^{i}\right)\right)+\frac{{\mu }_{hnf}}{{\mu }_{f}}\frac{1}{x{R}^{2}}\frac{\partial }{\partial x}\left(x\left(m+{\left(\frac{1}{R}\frac{\partial w}{\partial x}\right)}^{n-1}\right)\frac{\partial w}{\partial x}\right)\\ +\left(\frac{{\rho }_{hnf}}{{\rho }_{f}}\right){B}_{2}\left(\mathrm{cos}\left({c}_{2}{t}^{i}+\kappa \right)\right)+\left(\frac{{\left(\rho \gamma \right)}_{hnf}}{{\left(\rho \gamma \right)}_{f}}\right)Gr{\theta }_{j}^{i}sin\left(\alpha \right)-\frac{{\sigma }_{hnf}}{{\sigma }_{f}}{M}^{2}{w}_{j}^{i}\end{array}\right]$$

32 $${\theta }_{j}^{i+1}={\theta }_{j}^{i}+\frac{\Delta t}{\left(\frac{{\left(\rho {C}_{p}\right)}_{hnf}}{{\left(\rho {C}_{p}\right)}_{f}}\right)}\left[\begin{array}{c}\frac{1}{\mathrm{Pr}{R}_{e}}\left(\frac{{k}_{hnf}}{{k}_{f}}\right)\left(\frac{1}{{R}^{2}}\right)\left(\frac{{\partial }^{2}\theta }{\partial {x}^{2}}+\frac{1}{x}\frac{\partial \theta }{\partial x}\right)+\left(\frac{1}{{R}^{2}}\right)\frac{{N}_{R}}{\mathrm{Pr}{R}_{e}}\frac{{\partial }^{2}\theta }{\partial {x}^{2}}\\ +\left(\frac{{\sigma }_{hnf}}{{\sigma }_{f}}\right)\frac{{E}_{C}{M}_{a}^{2}}{{\mathrm{R}}_{e}}{w}_{j}^{i 2 }+\left(\frac{{\mu }_{hnf}}{{\mu }_{f}}\right)\frac{{E}_{C}}{{\mathrm{R}}_{e}}\left(\frac{1}{{R}^{2}}\right)\left(m+{\left(\frac{1}{R}\frac{\partial w}{\partial x}\right)}^{n-1}\right){\left(\frac{\partial w}{\partial x}\right)}^{2}\end{array}\right]$$

The boundary conditions are expressed in nodal form, as seen below.

33 $$\left.\begin{array}{ll}{w}_{j}^{1}={\theta }_{j}^{1}=0 & \quad {\text{a}}{\text{t}}t=0,\\ {w}_{N+1}^{i}={w}_{N}^{i},{\theta }_{N+1}^{i}={\theta }_{N}^{i}, & \quad {\text{a}}{\text{t}} \, x=0,\\ {w}_{N+1}^{i}=0,{\theta }_{N+1}^{i}=1, & \quad {\text{a}}{\text{t}}x=1.\end{array}\right\}$$

**Figure 2:**
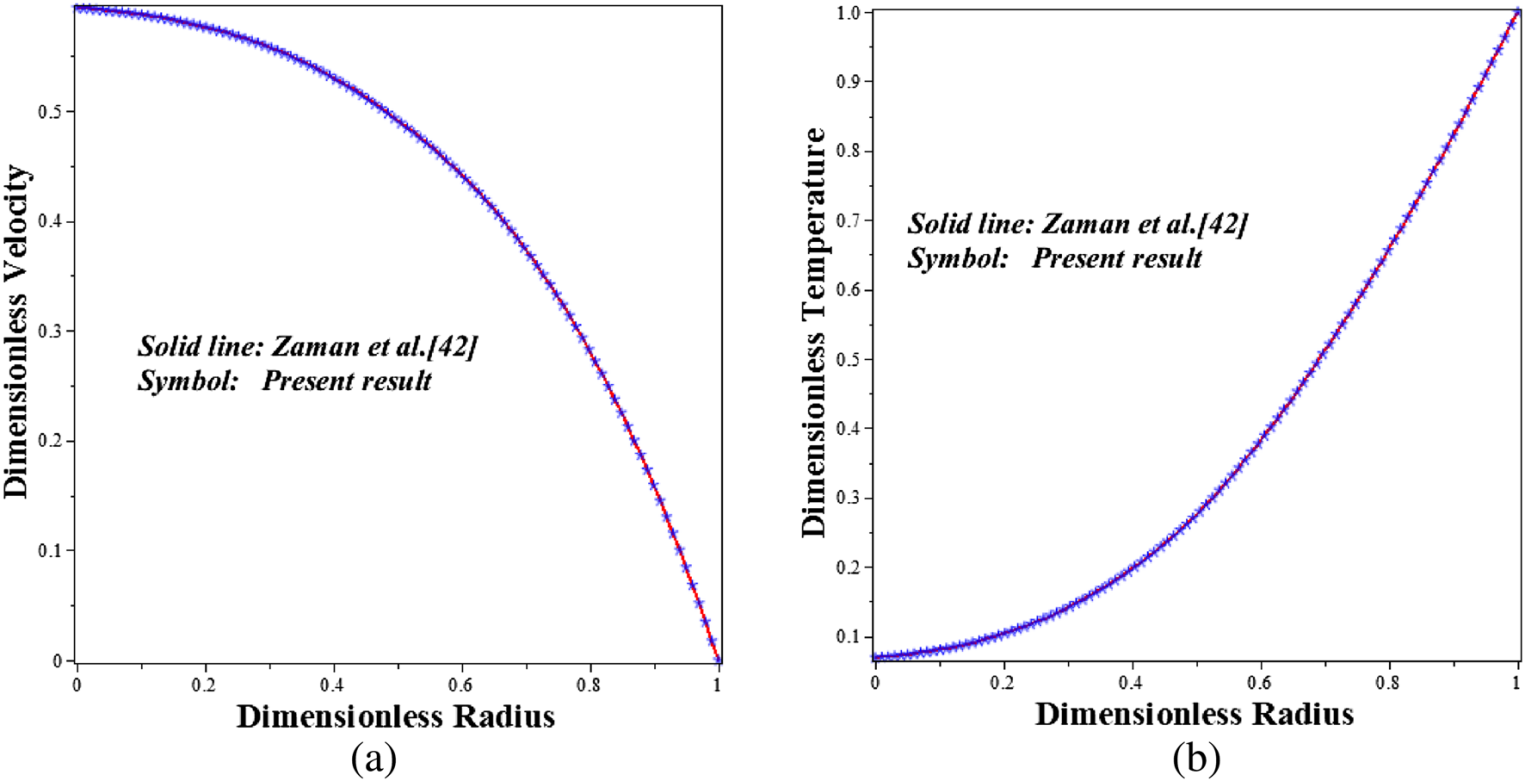
Present code is validated with Zaman et al.^42^ on (**a**) $Velocity$ and (**b**) $Temparature$ with $z=0.71, t=1.15$.

Due to the fact that the scheme's stability is wholly contingent on the time increment $\left(\Delta t\right)$ and the step size $\left(\Delta x\right)$, the $\Delta t$ = 0.0001 and $\Delta x$ = 0.025 are specified in order to address the stability constraint. These values have been shown to be appropriate for stability and convergence of the FTCS method in a number of investigations^36,38,41^. Table 2 displays numerical findings from this mathematical model, which have been validated against the published results of Ijaz and Nadeem^31^. This table demonstrates that the accepted FTCS code agrees very closely with the work of Ijaz and Nadeem^31^. In order to further validate the chosen numerical method employed to solve the current problem, the obtained numerical results for the axial velocity profile of blood flow are compared with the findings of Zaman et al.^42^ and Tripathi et al.^39^. These previous studies utilized the same modeling approach with the FTCS method, and the corresponding results are presented in Fig. 2 and Table 3. The comparison of these results demonstrates a significant level of agreement among them.

**Table 2:** Comparison of present code (FTCS) with Ijaz and Nadeem^31^ when $t=1.2, \; {\delta }^{*}=0.01, \; z=1.5,$ and $\phi =0.0$.

Radius $\left(x\right)$	Ijaz and Nadeem^31^	Present results
0.1	0.00	0.00
0.2	0.0317	0.0318
0.3	0.0418	0.0422
0.4	0.0420	0.0426
0.5	0.0366	0.0364
0.6	0.0283	0.0281
0.7	0.0191	0.0195
0.8	0.0109	0.0107
0.9	0.0052	0.0049
1.0	0.0039	0.0037

**Table 3:** A comparison is made between the present code (FTCS) and the study conducted by Zaman et al.^42^ and Tripathi et al.^31^ under the condition of $z=0.71, t=1.15$ on velocity.

Radius $\left(x\right)$	Zaman et al.^42^	Tripathi et al.^31^	Present results
0.0	0.5859	0.5881	0.589005298550675960
0.1	0.5829	0.5829	0.582898213609660765
0.2	0.5725	0.5725	0.572708824223326140
0.3	0.5540	0.5518	0.553959454552611747
0.4	0.5261	0.5215	0.525932428758457692
0.5	0.4864	0.4802	0.486570071001802918
0.6	0.4323	0.4257	0.433438445754765538
0.7	0.3605	0.3549	0.360834719368616783
0.8	0.2671	0.2637	0.268919798505806392
0.9	0.1483	0.1473	0.148154589828784192
1.0	0	0	0

## Response surface methodology (RSM)

The influence of critical flow field characteristics on heat transfer rate is best studied and optimized with the use of the response surface technique, the most effective statistical approach available. The central composite design focused on the face, developed by Box and Wilson^46^, is the main method in the RSM (CCD). In this study $Rd$, $M_{a}$ and $\phi_{1} + \phi_{2}$ on the heat transfer rate (Nu) are considered as the respective effective parameters and response variables. Table 4 depicts the assortment of selected effective parameters and their values. Correlations between response variables and active parameters take on their most basic form as follows, given a three-level, three-factor face-centered CCD-RSM:

$$\begin{aligned} Nu & ={\varpi }_{1}+{\varpi }_{2}{X}_{1}+{\varpi }_{3}{X}_{2}+{\varpi }_{4}{X}_{3}+{\varpi }_{5}{X}_{1}^{2}+{\varpi }_{6}{X}_{2}^{2}+{\varpi }_{7}{X}_{3}^{2}\\ & \quad +{\varpi }_{8}{X}_{1}{X}_{2}+{\varpi }_{9}{X}_{1}{X}_{3}+{\varpi }_{10}{X}_{2}{X}_{3}.\end{aligned}$$

where $\varpi$ regression coefficients.

**Table 4:** Key parameters, their symbols, and their levels for RSM.

Key factors	Symbols	Levels		
		− 1	0	1
		(Low)	(Medium)	(High)
$Rd$	X1	0	1	2
${M}_{a}$	X2	2	2.5	3
${\phi }_{1}+{\phi }_{2}$	X3	0	0.03	0.05

It should be noted that this model for experimentation involves 20 sets of runs, and the combinations for each set are shown in Table 5. In addition, this configuration takes into account six axial points, ten factorial points, and six centre points.

**Table 5:** Heat transfer rate experimental design and outcomes.

Order	Code values			Real values			Nu
	X1	X2	X3	$Rd$	${M}_{a}$	${\phi }_{1}+{\phi }_{2}$	
1	− 1	− 1	− 1	0	2	0	0.6343865838
2	1	− 1	− 1	2	2	0	0.3221672563
3	− 1	1	− 1	0	3	0	0.7788758703
4	1	1	− 1	2	3	0	0.3811767657
5	− 1	− 1	1	0	2	0.05	0.5849992150
6	1	− 1	1	2	2	0.05	0.2921378242
7	− 1	1	1	0	3	0.05	0.6931118918
8	1	1	1	2	3	0.05	0.3396071708
9	− 1	0	0	0	2.5	0.03	0.6738462984
10	1	0	0	2	2.5	0.03	0.3320529266
11	0	− 1	0	1	2	0.03	0.4465505877
12	0	1	0	1	3	0.03	0.5183164316
13	0	0	− 1	1	2.5	0	0.5134470831
14	0	0	1	1	2.5	0.05	0.4639040302
15	0	0	0	1	2.5	0.03	0.4848541319
16	0	0	0	1	2.5	0.03	0.4848541319
17	0	0	0	1	2.5	0.03	0.4848541319
18	0	0	0	1	2.5	0.03	0.4848541319
19	0	0	0	1	2.5	0.03	0.4848541319
20	0	0	0	1	2.5	0.03	0.4848541319

### Accuracy of the model

The ANOVA test and residual plots are used to evaluate the reliability of this experiment. The findings of the ANOVA for the wall-adjacent version of this design are shown in Table 6. Interactions at the linear, square, and two-way levels all have an effect on the statistical significance of separate variables. When employing the p-value, the significance of the independent parameter may be easily observed. A p-value of less than 0.05 indicates that these variables affect the rate of heat transfer. By evaluating the F and p values, we may determine the variance of the data and the regression model's significance. When the F value of the model terms is more than 1 and the p-value is less than 0.05, then the model terms are considered significant and are used in the analysis. As a result, the quadratic component of ${\phi }_{1}+{\phi }_{2}$, as well as the interaction terms of $Rd\times {\phi }_{1}+{\phi }_{2}$ and ${M}_{a}\times {\phi }_{1}+{\phi }_{2}$, are eliminated from the model Nu. It is also demonstrated in Table 6 that the statistical estimators for the simplified models of Nu are still statistically significant. Based on these regression coefficients, It can develop the following

**Table 6:** Analysis of variance for (ANOVA) for Nu.

Source	Degrees of freedom	Adjusted sum of squares	Adjusted mean square	F-value	P-value
Model	9	0.318640	0.035404	1864.27	0.000
Linear	3	0.314150	0.104717	5514.02	0.000
$Rd$	1	0.288838	0.288838	15,209.22	0.000
${M}_{a}$	1	0.018743	0.018743	986.97	0.000
${\phi }_{1}+{\phi }_{2}$	1	0.006569	0.006569	345.88	0.000
Square	3	0.001169	0.000390	20.52	0.000
$Rd$ $\times$ $Rd$	1	0.000863	0.000863	45.45	0.000
${M}_{a}\times {M}_{a}$	1	0.000022	0.000022	1.14	0.312
${\phi }_{1}+{\phi }_{2}\times {\phi }_{1}+{\phi }_{2}$	1	0.000008	0.000008	0.41	0.538
2-Way interaction	3	0.003483	0.001161	61.13	0.000
$Rd\times {M}_{a}$	1	0.002669	0.002669	140.54	0.000
$Rd$ $\times {\phi }_{1}+{\phi }_{2}$	1	0.000494	0.000494	26.01	0.000
${M}_{a}$ $\times {\phi }_{1}+{\phi }_{2}$	1	0.000320	0.000320	16.85	0.002
Error	10	0.000190	0.000019		
Lack-of-fit	5	0.000190	0.000038	*	*
Pure error	5	0.000000	0.000000		
Total	19	0.318830			

*Regression Equationin Uncoded Units*

34 $$\begin{aligned}Nu & =0.3002-0.12205{R}_{d}+ \text{ } 0.1918{M}_{a}+0.062 \, \left({\phi }_{1}\text{+}{\phi }_{2}\right)+0.01772{R}_{d}*{R}_{d} \\ & \quad -0.0112{M}_{a}*{M}_{a}-\text{ 2}.81\left({\phi }_{1}\text{+}{\phi }_{2}\right)*\left({\phi }_{1}\text{+}{\phi }_{2}\right)-0.03653 \, {R}_{d}*{M}_{a} \\ & \quad +0.3131{R}_{d}*\left({\phi }_{1}\text{+}{\phi }_{2}\right)-0.504{M}_{a}*\left({\phi }_{1}\text{+}{\phi }_{2}\right)\end{aligned}$$

**Figure 3:**
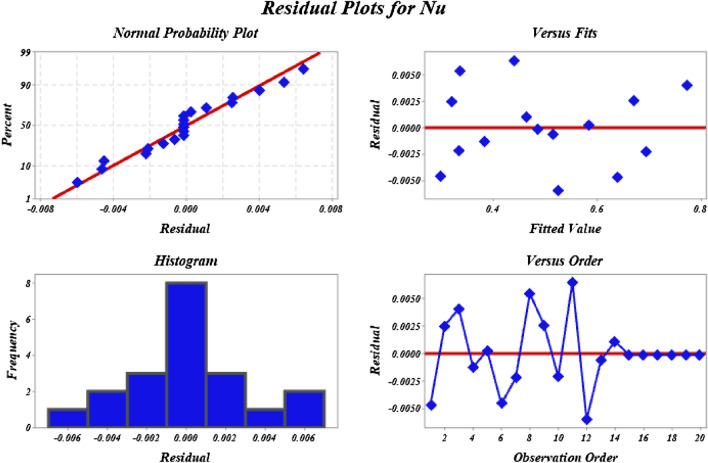
Residual plots for Nu.

Figure 3 shows residual plots for response values Nu, which are used to verify the reliability of the model. The points on the normal probability map line up perfectly with the theoretical expectation. The data exhibit minimal skewness and are mainly symmetrical, as seen by the histogram. This shows that the results obtained are representative. It can be seen from the residuals vs fitted plot that the highest residuals for Nu are around 0.0623. Additionally, S is 0.0043579, R^2^ is 99.94% adjusted- R^2^ is 99.89%, and R^2^ (Pred) is 99.16% tends to unity, achieving the best fitted model. These details support the conclusion that the selected model is reliable and well-fitting.

### Response surface analysis

**Figure. 4:**
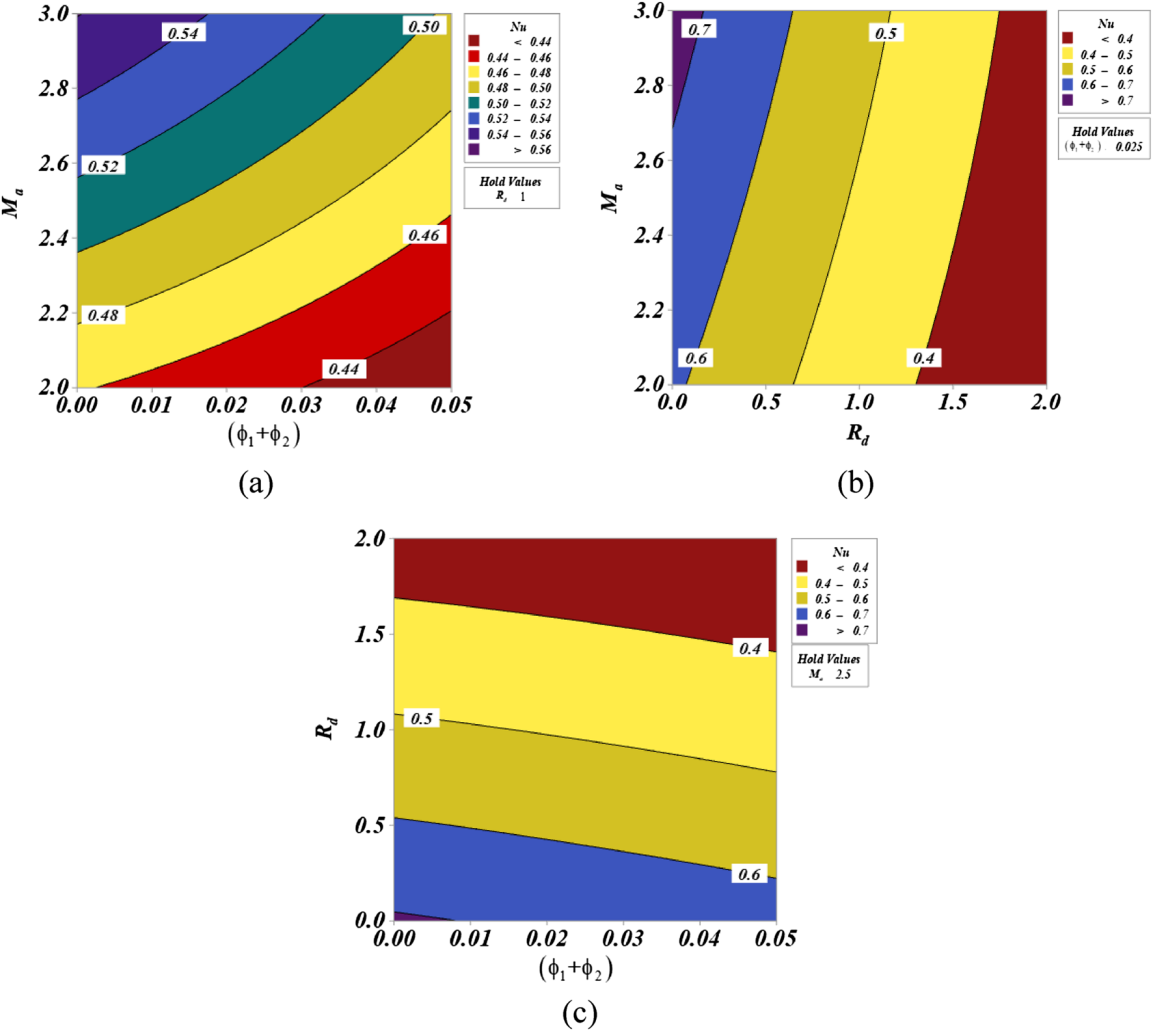
Contour plot for the various interactive parameters.

Figure 4 explores the contour plots projected to investigate the impacts of effective parameters ($Rd$,${M}_{a}$, & ${\phi }_{1}+{\phi }_{2}$) on the responses Nu. Figure 4a–c depicts the effect of (a) interaction ${\phi }_{1}+{\phi }_{2}$ with ${M}_{a}$, (b) interaction $Rd$ with ${M}_{a}$, and (c) interaction ${\phi }_{1}+{\phi }_{2}$ with $Rd$ on Nu. It is important to note that the interaction between two components is carried out at all levels, and the middle value of the third parameter is held as a hold value. The heat transfer rate is increased by the lower ${M}_{a}$ and ${\phi }_{1}+{\phi }_{2}$ values. In the cases of $Rd$ and ${M}_{a}$, the similar nature is shown. As $Rd$ and ${\phi }_{1}+{\phi }_{2}$ are increased, the rate of heat transfer increases.

### Sensitivity analysis

To find out how much variation there is in the heat transfer rate, we turn to the independent parameters. The following sensitivity functions are used to evaluate the growth or decline of responses (Nu) with regard to $Rd$,${M}_{a}$, & ${\upphi }_{1}+{\upphi }_{2}$ from Eq. (34):

$$\frac{\partial Nu}{\partial {\text{R}}_{\text{d}}}= \text{ } -0.12205+\text{0.03544}{X}_{1}-0.03653{X}_{2}+0.3131{X}_{3},$$

$$\frac{\partial Nu}{\partial {\text{M}}_{\text{a}}}=0.1918-0.03653{X}_{1}-0.0224{X}_{2}-0.504{X}_{3},$$

$$\frac{\partial Nu}{\partial \left({\upphi }_{1}\text{+}{\upphi }_{2}\right)}=0.0620+0.3131{X}_{1}-0.5040{X}_{2}-5.620{X}_{3}.$$

**Figure 5:**
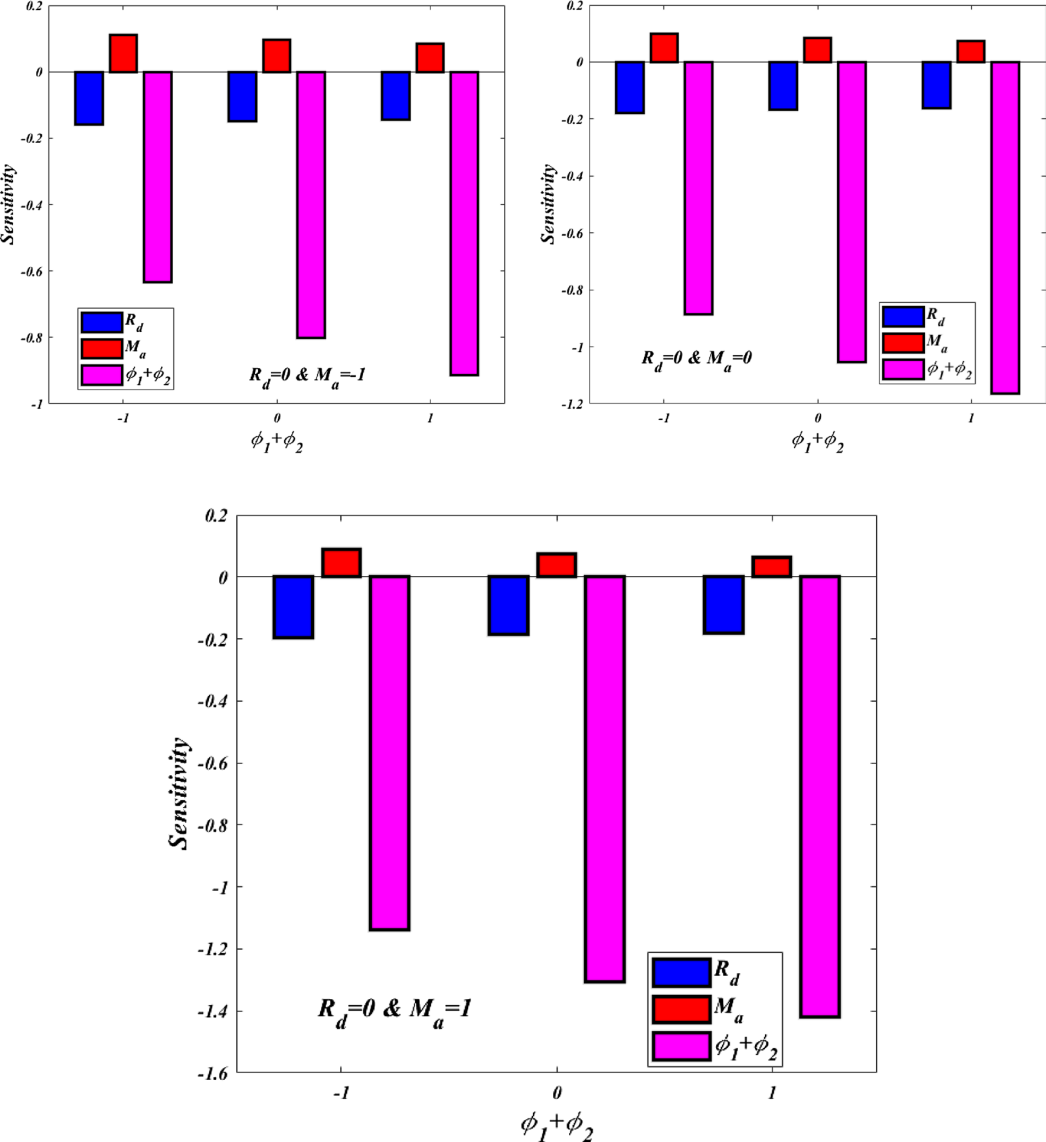
Medium-${\text{R}}_{\text{d}}$ sensitivity of operating parameters.

Importantly, the sensitivity is determined for the middle value of X1, whereas Table 7 shows all possible values for the other two factors. To investigate the sensitivity of independent factors, as shown in Fig. 5. It is obvious that ${M}_{a}$ is the positive side and that $Rd$ and ${\upphi }_{1}+{\upphi }_{2}$ have a negative sensitivity.

**Table 7:** Response sensitivity at the medium value of ${\text{R}}_{\text{d}}$.

Order	${X}_{1}$	${X}_{2}$	${X}_{3}$	$\frac{\partial Nu}{\partial {X}_{1}}$	$\frac{\partial Nu}{\partial {X}_{2}}$	$\frac{\partial Nu}{\partial {X}_{3}}$
1	0	− 1	− 1	− 0.15967	0.11047	− 0.6329
2	0	− 1	0	− 0.150277	0.09535	− 0.80150
3	0	− 1	1	− 0.144015	0.08527	− 0.91390
4	0	0	− 1	− 0.177935	0.09927	− 0.88490
5	0	0	0	− 0.168542	0.08415	− 1.05350
6	0	0	1	− 0.162280	0.07407	− 1.16590
7	0	1	− 1	− 0.19620	0.08807	− 1.1369
8	0	1	0	− 0.186807	0.07295	− 1.30550
9	0	1	1	− 0.180545	0.06287	− 1.41790

## Results and discussion

The purpose of the current investigation is to demonstrate the time-dependent sensitivity analysis and entropy generation of Sisko blood hybrid nanofluid flow containing gold-tantalum nanoparticles in a slanted artery with composite stenosis. This section uses pictures to show how various factors effects on Infinite shear rate viscosity parameter $\left(m=1.5,\hspace{0.17em}2.0,\hspace{0.17em}2.5,\hspace{0.17em}3.0\right)$, Hartmann number $\left({M}_{a}=2.0,\hspace{0.17em}2.4,\hspace{0.17em}2.8,\hspace{0.17em}3.2\right)$ ,angle parameter $\left(\mathrm{\alpha }{=}^{\uppi }{/}_{3},\hspace{0.17em}{\hspace{0.17em}}^{\uppi }{/}_{4},\hspace{0.17em}{\hspace{0.17em}}^{\uppi }{/}_{5},\hspace{0.17em}{\hspace{0.17em}}^{\uppi }{/}_{6}\right)$, nanoparticle volume fraction $\left({\upphi }_{1}+{\upphi }_{2}=0.0,\hspace{0.17em}0.01,\hspace{0.17em}0.03,\hspace{0.17em}0.05\right)$, amplitude of body acceleration $\left({B}_{2}=0.5,\hspace{0.17em}1.0,\hspace{0.17em}1.5,\hspace{0.17em}2.0\right)$ Reynolds number $\left({R}_{e}=0.5,\hspace{0.17em}1.0,\hspace{0.17em}1.5,\hspace{0.17em}2.0\right)$, Grashof number $\left({G}_{r}0.1,\hspace{0.17em}0.2,\hspace{0.17em}0.3,\hspace{0.17em}0.4\right)$ and Thermal radiation $\left({R}_{d}=0.0,\hspace{0.17em}0.5,\hspace{0.17em}1.0,\hspace{0.17em}1.5\right)$ on velocity, temperature non-dimensional, entropy generation $\left({N}_{G}\right)$, flow rate, Resistance to flow, Wall shear stress and Nusselt number.

**Figure 6:**
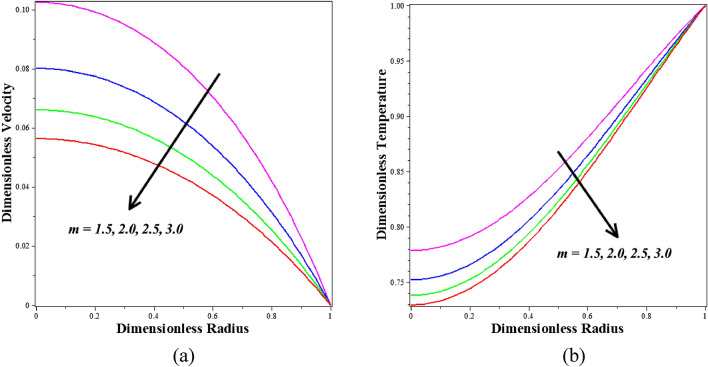
Different values of $m$ on (**a**) velocity and (**b**) temperature.

Dimensionless profiles of velocity and temperature for different values of the infinite shear rate viscosity $\left(m\right)$ are shown in Fig. 6a,b, respectively. Figure 6a demonstrates that improving Infinite shear rate viscosity $\left(m\right)$ tend to decrease the *Ta-Au* /blood hybrid nanofluid velocity profile . The *Ta-Au* /blood hybrid nanofluid temperature profile exhibits roughly the same behavior. Physically, the higher blood viscosity caused by the stronger infinite shear rate viscosity exponent causes a decrease in the shear rate, which slows down the velocity and temperature.

**Figure 7:**
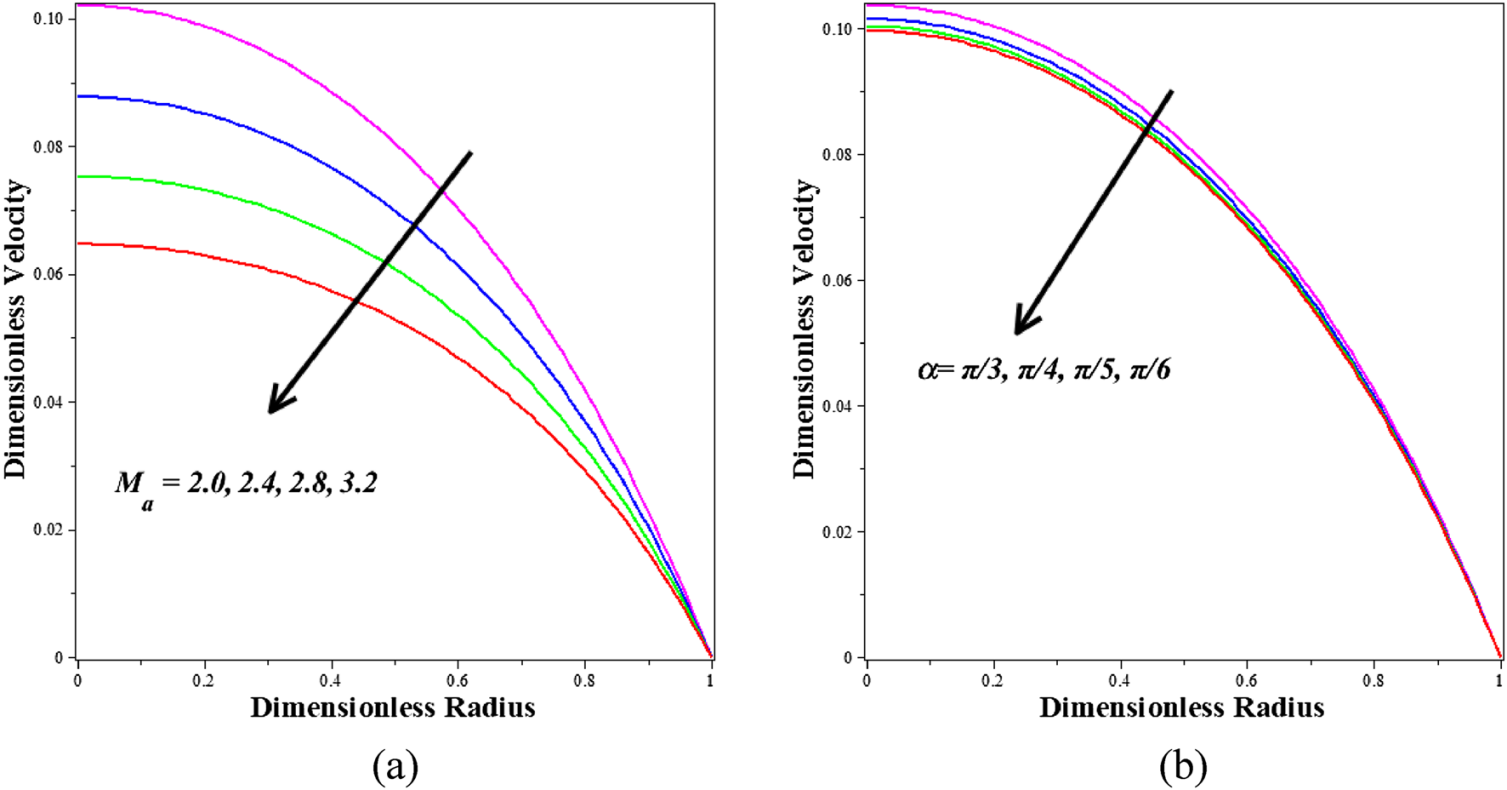
Different values of (**a**) ${M}_{a}$ and (**b**) $\alpha$ on Velocity.

Figure 7a,b depict the effect of various active features of the Hartmann number $\left({M}_{a}\right)$ and angle parameter $\left(\alpha \right)$ on the velocity profile. Figure 7a illustrates the decrease in the Hartmann number $\left({M}_{a}\right)$ in the velocity profile of blood-based *Ta-Au* hybrid nanofluids. It becomes evident that an increased value of M (associated with a more potent Lorentzian drag force) noticeably hinders the blood flow in the core zone, which is the region around the centerline of the artery. In the case of M = 0, representing a radial magnetic field of zero, the electrically non-conducting blood is obtained. For M = 2, both the magnetic and viscous hydrodynamic forces are equivalent. The blood flow is effectively regulated and damped by a stronger magnetic field, enabling enhanced control of biomedical procedures through a non-intrusive approach, such as employing an external magnetic field. This parameter is also significant for adjusting the velocity of the fluid. It has been discovered that reducing the velocity profile will increase the values of angle parameter $\left(\alpha \right)$. The effect of varying the angle parameter $\left(\alpha \right)$ on the velocity of a Ta-Au hybrid nanofluid based on blood is shown in Fig. 7b. It's clear that the blood velocity drops down precipitously as the readings go higher. In this model, the thermal buoyancy component is accounted for in the momentum equation. Furthermore, decreasing blood velocity is another benefit of enhancing the buoyancy effect.

**Figure 8:**
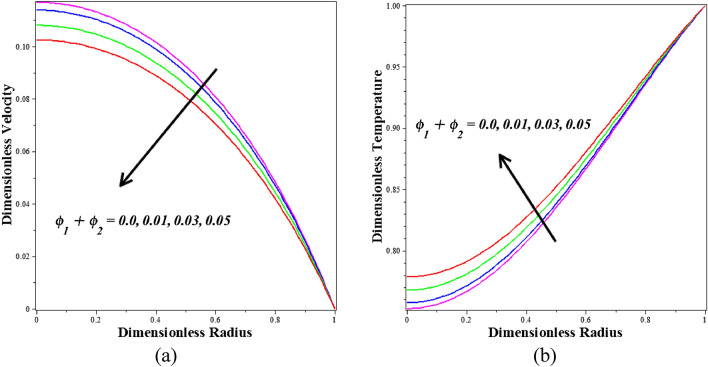
Different values of ${\phi }_{1}+{\phi }_{2}$ on (**a**) velocity (**b**) temperature.

**Figure 9:**
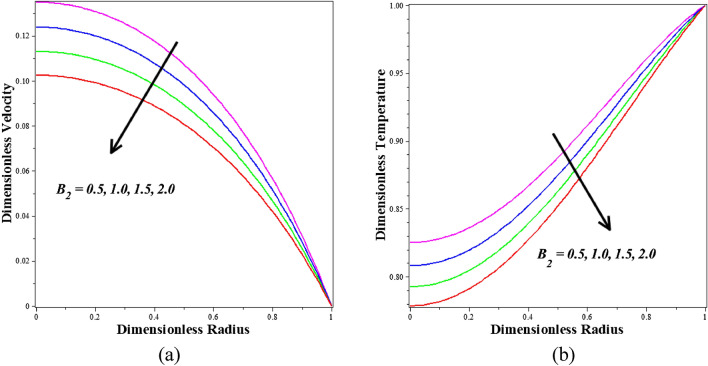
Different values of ${B}_{2}$ on (**a**) velocity and (**b**) temperature.

Figure 8a,b describe how the nanoparticle volume fraction $\left({\phi }_{1}+{\phi }_{2}\right)$ is influenced by velocity and temperature profiles. It is observed that blood-based *Ta-Au* hybrid nanofluids velocity profile is reducing for enhancing the $\left({\phi }_{1}+{\phi }_{2}\right)$. It is detected that blood-based *Ta-Au* hybrid nanofluids temperature profiles expanding for improving the values of $\left({\phi }_{1}+{\phi }_{2}\right)$. The absence of indicates that there are no nanoparticles suspended in the blood's base liquid. A significant quantity of heat energy is added to the blood as the volume fraction of nanoparticles rises. Nanoparticles often exhibit better thermal properties than base fluids. Additionally, examples of developing nanoparticles. Figure 9a,b show the dimensionless steady state period at which the maximum velocity is attained, as well as the dimensionless velocity and temperature profiles for different values of the dimensionless amplitude of the body acceleration. Based on the graphs, it can be deduced that as the body accelerates more rapidly, the amplitude of the velocity and temperature profiles diminishes.

**Figure 10:**
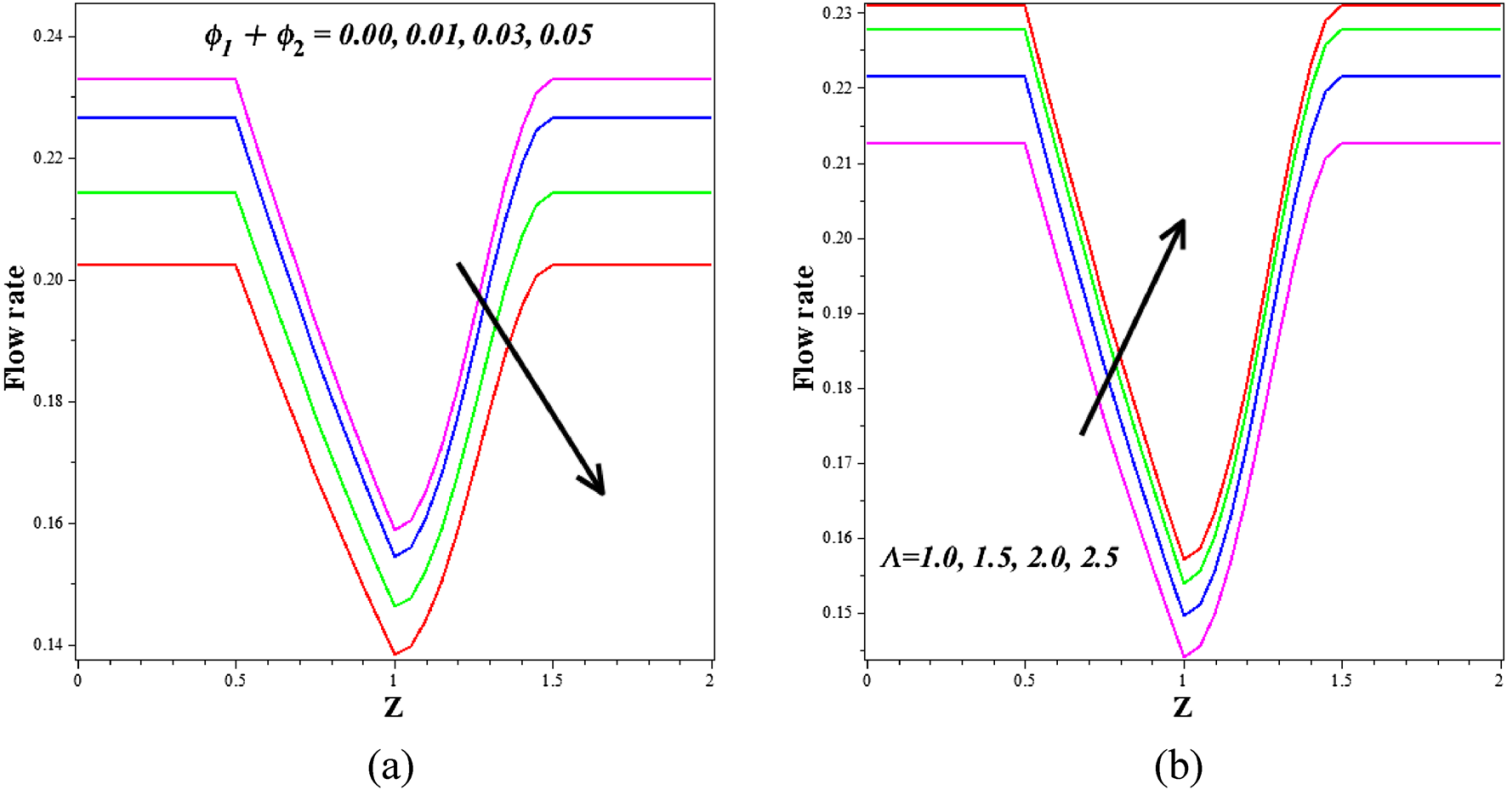
Different values of (**a**) ${\phi }_{1}+{\phi }_{2}$ and (**b**) $\Lambda$ on flow rate.

**Figure 11:**
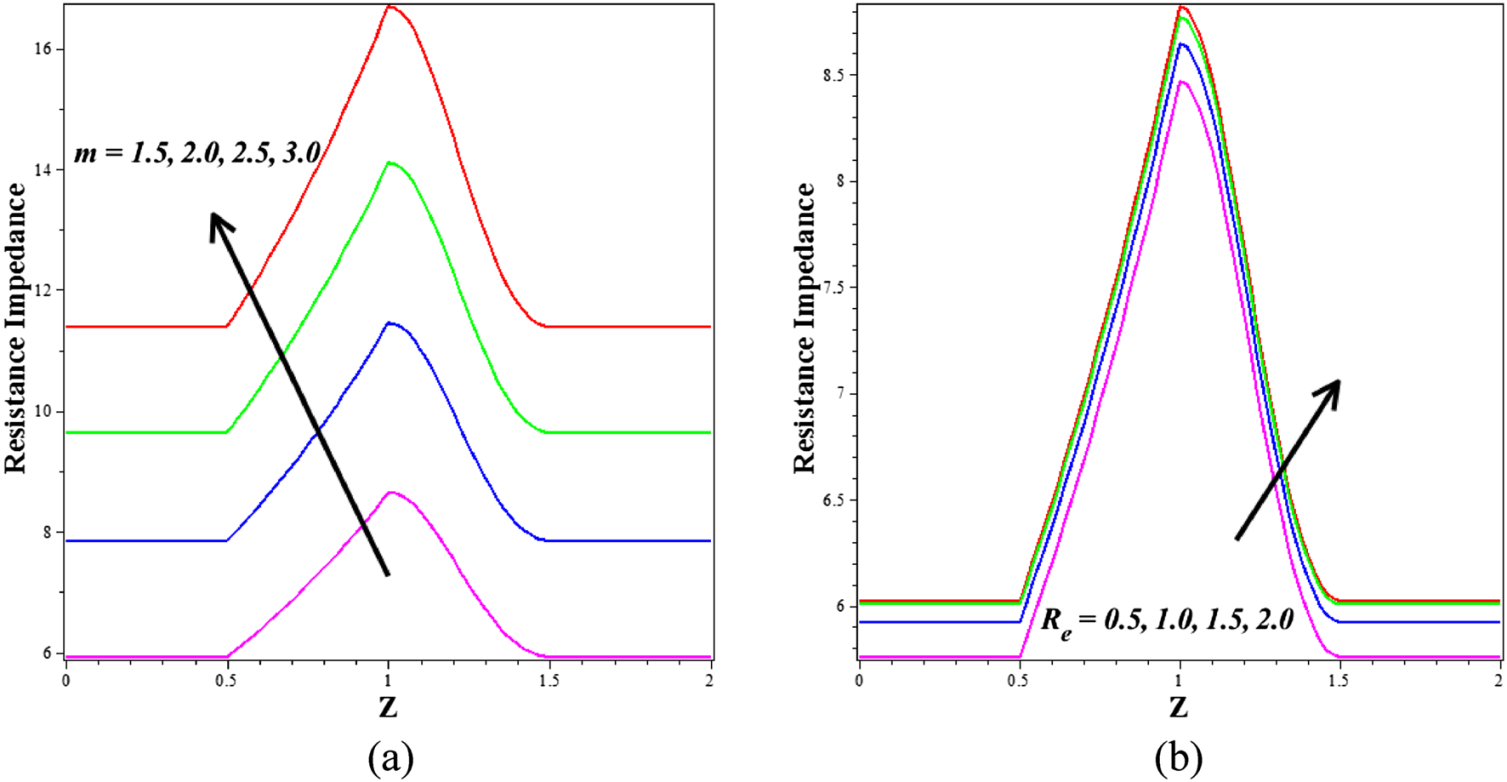
Different values of (**a**) $m$ and (**b**) ${R}_{e}$ on resistance to flow.

The effect of $\left({\phi }_{1}+{\phi }_{2}\right)$ on dimensionless flow rate in the stenotic zone is shown in Fig. 10a. This graph demonstrates that when $\left({\phi }_{1}+{\phi }_{2}\right)$ increases, volume flow rate falls. Additionally, Fig. 10b illustrates the flow rate's Womersley number nature, i.e., how the flow rate rises in response to an improvement in the values of the Womersley number. The most typical method for organs to control blood flow is through changes in resistance. The relationship between flow rate and impedance is inverse. The consequence of the Infinite shear rate viscosity $\left(m\right)$ and Reynolds number $\left({R}_{e}\right)$ on resistance to flow is represented in Fig. 11a,b. Figure 11a shows that the resistance to flow enlargements as the infinite shear rate viscosity $\left(m\right)$ increases. Physically, the higher blood viscosity caused by, the stronger infinite shear rate viscosity exponent causes an increase in the shear rate, which is resistance to fluid flow. The resistance to flow enlarges when the Reynolds number $\left({R}_{e}\right)$ rises, as seen in Fig. 11b. The outcomes of the earlier axial velocity computations are corroborated by a continuous suppression of flow rate as Re values grow from 1 to 5. Because inertial effects become more noticeable at higher Reynolds numbers, flow retardation and decreased flux are the primary consequences. The resistive impedance profiles increase as expected due to the dominance of viscous forces over inertial forces.

**Figure 12:**
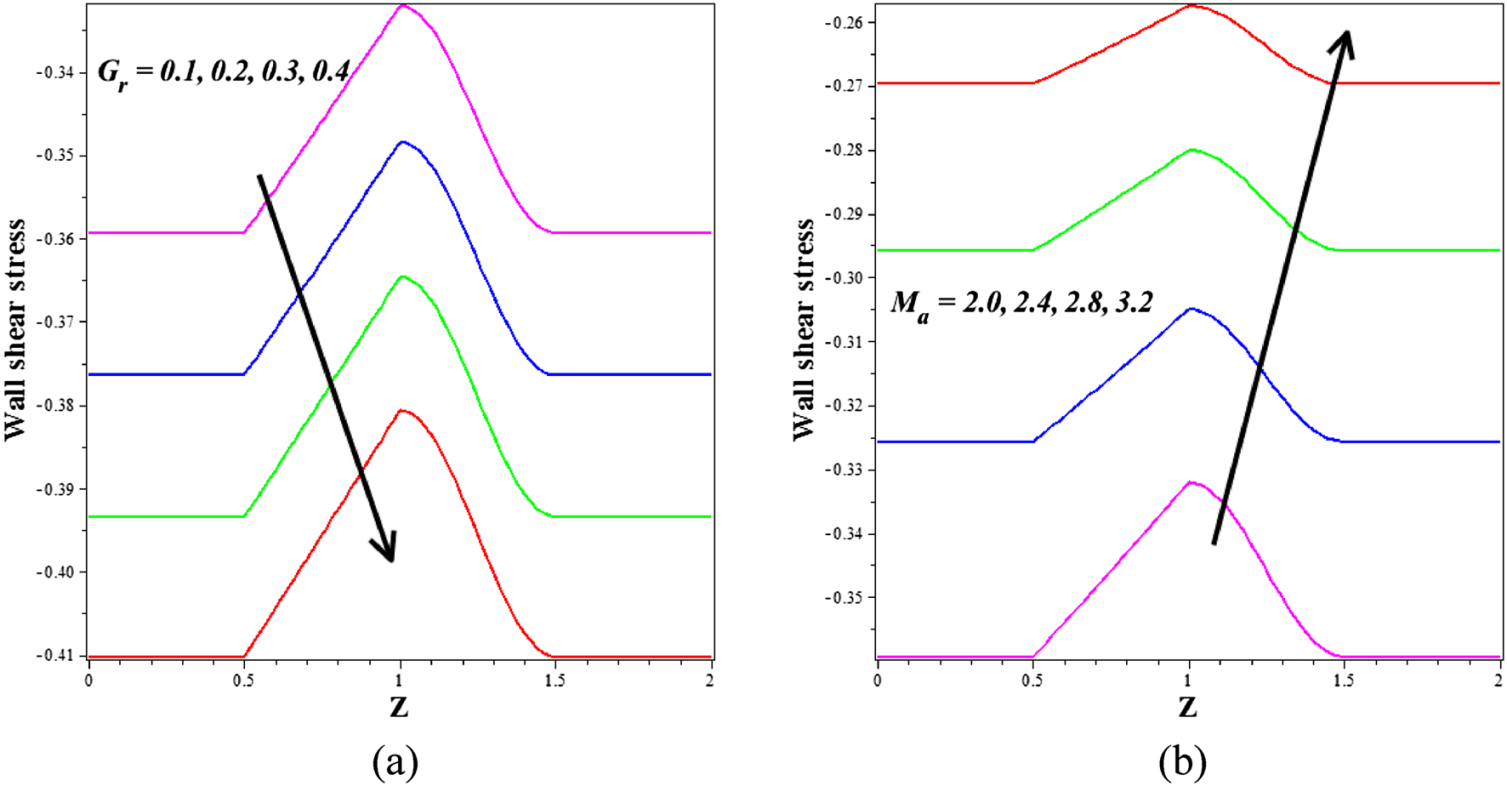
Different values of (**a**) ${G}_{r}$ and (**b**) ${M}_{a}$ on wall shear stress.

**Figure 13:**
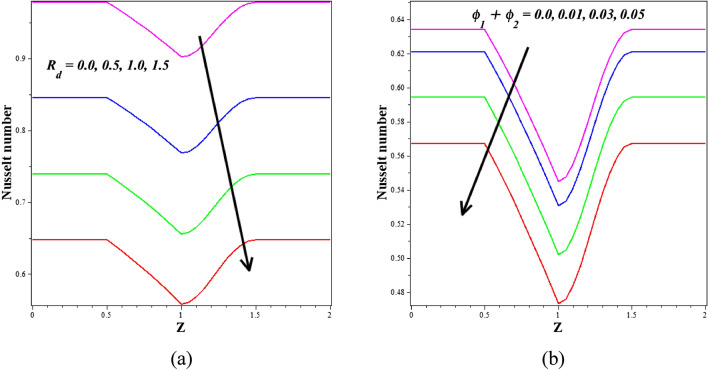
Different values of (**a**) ${R}_{d}$ and (**b**) ${\phi }_{1}+{\phi }_{2}$ on Nusselt number.

**Figure 14:**
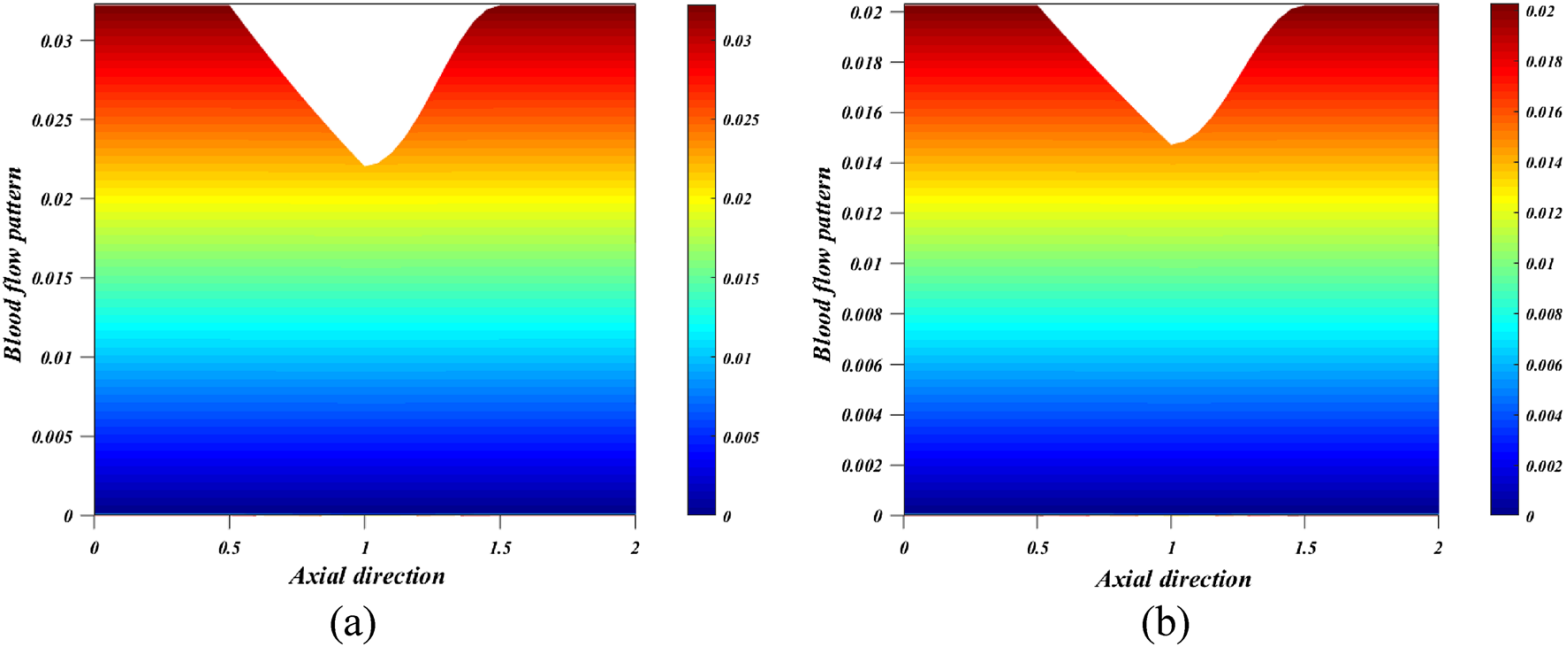
Different values of $M$ (**a**) $M=2$ (**b**) $M=3.2$ on blood flow pattern.

**Figure 15:**
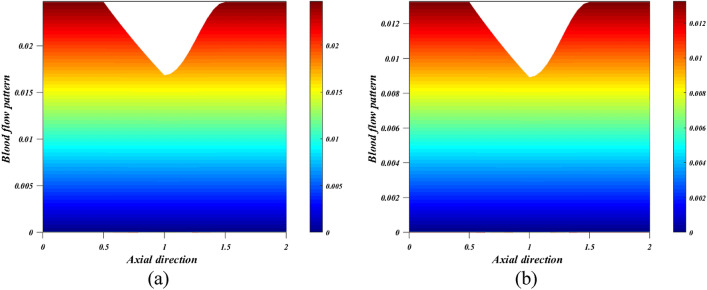
Different values of $m$ (**a**) $m=2$ (**b**) $m=4$ on blood flow pattern.

**Figure 16:**
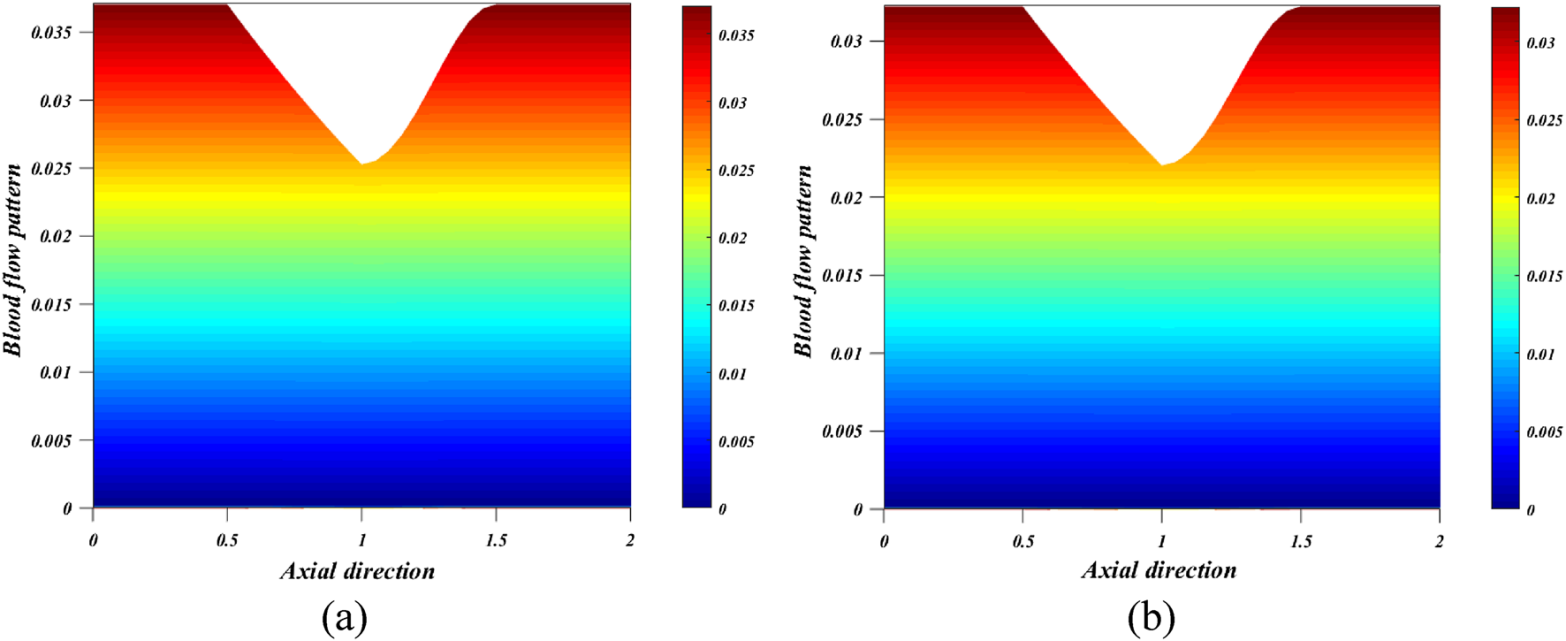
Different values of ${\phi }_{1}+{\phi }_{2}$ (**a**) ${\phi }_{1}+{\phi }_{2}=0$ (**b**) ${\phi }_{1}+{\phi }_{2}=0.05$ on blood flow pattern.

When the wall exerts a force per unit area on the fluid in the direction perpendicular to the local tangent plane, we call this "wall shear stress" in the context of arterial blood flow. The atherogenic process is controlled by pulsatile blood flow, which generates wall shear stress. Figures 12a,b depict the effect of various active features of the Grashof number $\left({G}_{r}\right)$ and Hartmann number $\left({M}_{a}\right)$ on the wall shear stress. The ratio between buoyancy and viscous force is called the Grashof number $\left({G}_{r}\right)$. Different nanoparticle species diffuse at different rates due to differences in the thermal buoyancy force caused by the quantity of nanoparticles in circulation. Additionally, blood flow resistance is reduced due to this occurrence, which leads to an acceleration and a consequent fall in wall shear stress profiles. Due to the generation of the Lorentz force, an opposing force to the flow that increases velocity and, in turn, increases wall shear stress, there is a rise in $\left({M}_{a}\right)$ values as well as an increase in wall shear stress. Figure 13a,b is plotted for investigating the consequence of thermal radiation $\left({R}_{d}\right)$ and nanoparticle volume fraction $\left({\phi }_{1}+{\phi }_{2}\right)$ aspects in the heat transfer coefficient. Figure 13a illustrates how $\left({R}_{d}\right)$ varies in relation to the rate of heat transmission. The heat transmission rate is decreased by $\left({R}_{d}\right)$ settings according to this figure. It is observed that blood-based *Ta-Au* hybrid nanofluids Nusselt number is reducing for enhancing the $\left({\phi }_{1}+{\phi }_{2}\right)$ is shown in Fig. 13b. The absence of indicates that there are no nanoparticles suspended in the blood's base liquid. The impacts of the magnetic field, the viscosity parameter, and the nanoparticle volume fraction on the blood flow pattern is explored in Figs. 14, 15, 16 (a) and (b). It is seen from these figures that the blood flow pattern is lessened with the effects of all three parameters.

**Figure 17:**
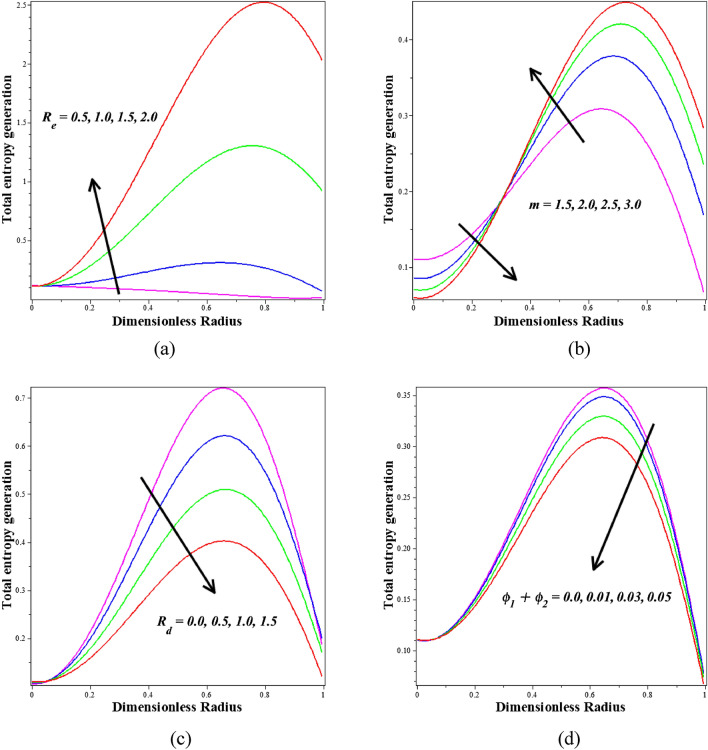
Different values of (**a**) ${R}_{e}$ (**b**) $m$ (**c**) ${R}_{d}$ and (**d**) ${\phi }_{1}+{\phi }_{2}$ on Total entropy generation.

Figure 17a–d manifested the total entropy generation has been examined Reynolds number $\left({R}_{e}\right)$, infinite shear rate viscosity $\left(m\right)$, thermal radiation $\left({R}_{d}\right)$ and nanoparticle volume fraction $\left({\phi }_{1}+{\phi }_{2}\right)$ through numerical integration. Figure 17a demonstrates that improving Reynolds numbers $\left({R}_{e}\right)$ tend to increase the entropy generation. The ratio of inertial and viscous forces is identified as the Reynolds number $\left({R}_{e}\right)$. As a result, as $\left({R}_{e}\right)$ increases, the viscous forces tend to decrease and the entropy generation increases. Figure 17b displays how the entropy generation profile and infinite shear rate viscosity $\left(m\right)$ are inversely connected. This graph demonstrates that the entropy generation decreases at the lower values and increases with higher values of the infinite shear rate viscosity $\left(m\right)$. Physically, the higher blood viscosity caused by the stronger infinite shear rate viscosity exponent causes a decrease in the shear rate. Figure 17c illustrates how raising the thermal radiation $\left({R}_{d}\right)$ tends to reductions the generation of entropy. It is observed that blood-based *Ta-Au* hybrid nanofluids entropy generation is reducing for enhancing the $\left({\phi }_{1}+{\phi }_{2}\right)$ is shown in Fig. 17d.

## Conclusion

The purpose of this study is to demonstrate the fluid flow, heat transfer, and entropy generation of Sisko blood hybrid nanofluid flow containing gold-tantalum nanoparticles in a slanted artery with composite stenosis in the presence body acceleration, thermal radiation and Hartmann number. The volume fraction model is used to demonstrate the features of a hybrid nanofluid. The controlling flow equations are thought to be simplified by the mild stenosis approximation. To solve the nondimensionalized flow equations, the finite-difference method is employed. The significant parameters of temperature non-dimensional, entropy generation $\left({N}_{G}\right)$, flow rate, Resistance to flow, Wall shear stress and Nusselt number are projected using two-dimensional graphs. The following are the model's primary findings:

- Growing infinite shear rate viscosity leads to a decrease in the blood nanofluid velocity and temperature.
- The addition of gold-tantalum nanoparticles to the base fluid promotes a temperature that is higher than that of the Sisko blood base fluid.
- Intensifying radiation and increasing the nanoparticle volume fraction both slow down the rate of heat transfer.
- A significant decrease is computed in the total entropy generation with greater values of thermal radiation and nanoparticle volume fraction.
- As the magnetic field increases, the heat transfer rate increases in the absence of thermal radiation.
- Highly negative sensitivity for nanoparticle volume fraction is observed when the higher level of magnetic field and nanoparticle volume fraction.
- The high coefficient of determination indicates that the quadratic model of the response surface approach is a good fit for the lowered heat transfer rate.
- The nanoparticle volume fraction and thermal radiation are negatively sensitive to the heat transfer rate, while the magnetic field aspect has positively sensitive.

The current simulations provide an opportunity for further exploration of the electro-osmotic characteristics of blood flow and the investigation of tri-hybrid nanoparticles that hold potential for nano-drug delivery. These areas of interest extend to nanomedicine, targeted drug delivery, diverse industrial applications, and the theoretical investigation of hemodynamic effects in stenotic arteries. The findings from this research contribute to the advancement of understanding hybrid nanoparticle formation and its implications in various fields. Moreover, it paves the way for future innovative approaches in addressing conditions such as atherosclerosis and cancer by comprehensively studying different blood flow models in composite stenosed arteries.

## Data Availability

All data generated or analyzed during this study are included in this published article.
